# Ravens parallel great apes in physical and social cognitive skills

**DOI:** 10.1038/s41598-020-77060-8

**Published:** 2020-12-10

**Authors:** Simone Pika, Miriam Jennifer Sima, Christian R. Blum, Esther Herrmann, Roger Mundry

**Affiliations:** 1 Comparative BioCognition, Institute of Cognitive Science, University of Osnabrück, Artilleriestrasse 34, 49076 Osnabrück, Germany; 2 Research Group “Evolution of Communication”, Max Planck Institute for Ornithology, Seewiesen, Germany; 3 Department of Behavioral and Cognitive Biology, University of Vienna, Vienna, Austria; 4 Research Group “Human Origins of Self-Regulation”, Max Planck Institute for Evolutionary Anthropology, Leipzig, Germany; 5 Department of Psychology, Centre for Comparative and Evolutionary Psychology, University of Portsmouth, Portsmouth, UK; 6 Interim Group Primatology, Max Planck Institute for Evolutionary Anthropology, Leipzig, Germany

**Keywords:** Evolutionary developmental biology, Human behaviour, Social evolution

## Abstract

Human children show unique cognitive skills for dealing with the social world but their cognitive performance is paralleled by great apes in many tasks dealing with the physical world. Recent studies suggested that members of a songbird family—corvids—also evolved complex cognitive skills but a detailed understanding of the full scope of their cognition was, until now, not existent. Furthermore, relatively little is known about their cognitive development. Here, we conducted the first systematic, quantitative large-scale assessment of physical and social cognitive performance of common ravens with a special focus on development. To do so, we fine-tuned one of the most comprehensive experimental test-batteries, the Primate Cognition Test Battery (PCTB), to raven features enabling also a direct, quantitative comparison with the cognitive performance of two great ape species. Full-blown cognitive skills were already present at the age of four months with subadult ravens’ cognitive performance appearing very similar to that of adult apes in tasks of physical (quantities, and causality) and social cognition (social learning, communication, and theory of mind). These unprecedented findings strengthen recent assessments of ravens’ general intelligence, and aid to the growing evidence that the lack of a specific cortical architecture does not hinder advanced cognitive skills. Difficulties in certain cognitive scales further emphasize the quest to develop comparative test batteries that tap into true species rather than human specific cognitive skills, and suggest that socialization of test individuals may play a crucial role. We conclude to pay more attention to the impact of personality on cognitive output, and a currently neglected topic in Animal Cognition—the linkage between ontogeny and cognitive performance.

## Introduction

How intelligence evolved still remains one of science’s greatest mysteries. However, the past few years have seen two major and interrelated streams of research, one focusing on the evolution of the brain, and the other one pinpointing similarities and differences in behaviour (e.g.^1–5^). The majority of research interest has been devoted to the primate order^6–9^, thereby incorporating information about the phylogenetic relationships between species as well as presumed selective pressures acting upon the development of cognitive skills. One of the most comprehensive experimental studies tapping into the wide spectrum of physical and social cognitive domains has been carried out by Herrmann and colleagues^10^. They designed a test battery to compare the cognitive skills of human children, and two of our closest living relatives, chimpanzees (*Pan troglodytes*), and orangutans (*Pongo pygmaeus*). Two and a half year old children and chimpanzees (mean age: 10 years) showed very similar cognitive performance for dealing with the physical world, suggesting that human children’s physical cognitive skills are still equivalent to those of our last common ancestor some 6 million years ago^10^. In stark contrast, the children outperformed both great ape species in tasks dealing with the social world (see for similar results on bonobos *Pan paniscus*^11^). The authors argued that these results provide no support for the general intelligence hypothesis^12^ predicting that human cognition differs from that of apes only in general cognitive processes (such as memory, learning, or perceptual processing). Rather, human infants’ social cognitive skills are already on a species-specific cognitive path, involving a species-specific set of social-cognitive skills for participating and exchanging knowledge in cultural groups^10^. It thus has been suggested that crucial developments in skills of human social-cultural cognition may have occurred only post-*erectus*, in support of especially complex forms of collaborative activity, such as hunting or gathering, supported by special skills of communication and social learning^13^. These abilities possibly developed from earlier evolved primate skills of social cognition, communication and learning in general that nonhuman primates display in their everyday interactions to cope with the challenges of their social environments^14–17^. In line with this, Schmitt and colleagues^18^ recently showed that the performance of individuals of two Old World monkey species (long-tailed macaques *Macaca fascicularis*; olive baboons *Papio anubis*) tested with the PCTB^10^ was largely comparable with those of individuals of the two great ape species.

Predominant theories propose that the distinctive aspects of primate cognition evolved mainly in response to the cognitive challenges of the ecological environment (the Ecological Intelligence hypothesis^19–21^), or the challenges of the social environment including the need to form and maintain social bonds, cooperation, track third-party relationships, and anticipate the behaviour of conspecifics (the Social Intelligence hypothesis^14, 22^). The latter hypothesis, especially, inspired an enormous research interest with studies showing that measures of social complexity and/or competence indeed correlate with neocortex size^23,24^. The neocortex is a set of layers of the mammalian cerebral cortex, and is involved in higher-order brain functions such as sensory perception, cognition, generation of motor commands, spatial reasoning, and language^25^. However, it has been argued that size per se might not be the critical factor but the modularity, interconnectedness and neuron numbers of different brain areas^4,26^. For instance, primates have more neurons than non-primate mammals with identical brain sizes, and humans show the highest neuron numbers, totaling 86 billion neurons^27^. Others argued that attempts to link brain size to function are problematic due to the choice of variables being entered in the analyses, and the problems associated with multiple correlations^28^. In addition, the nature of social relationships seems to highly impact upon brain size, irrespective of group stability and social dynamics. For instance, species forming long-term relationships and/or partnerships tend to have bigger brains than species engaging in short-term or seasonal relationships only (^17^, but see^29^). Hence, interacting with particular individuals over time—rather than interacting with many individuals and/or over limited periods only—seems to be a highly cognitively challenging enterprise^30^. Although the Social Intelligence hypothesis was originally applied to non-human primates only^14^, the idea has recently inspired a lot of research studies in other taxa showing sophisticated social relationships and/or long-term bonds (e.g.^31,32^). Due to their unique combination of fission–fusion dynamics and strong long-term relationships^30^, members of a distinct songbird family—corvids—have been of special interest to researchers wishing to unravel the puzzle of the evolution of intelligence^33, 34^. It has been suggested that the evolutionary lines of mammals and birds separated approximately 300 million years ago^35^. This extremely long period of parallel evolution is apparent in the brain organization of the two classes^36^, with the subpallial territory, a part of the cerebrum, showing a strikingly similar organization in mammals and birds^37^. In contrast, the evolutionary trajectory of the pallium is less clear^36^. While in mammals it is mainly made up by the six-layered cortex, the avian pallium is characterized by the lack of any laminar structure^38^. However, Stacho and colleagues^39^ recently described that the avian pallium has a cyto-architectonic organization that is reminiscent of the mammalian cortex. Furthermore, passerine but also parrot brains have twice as many neurons as primate brains of the same size^4^, and the neuronal density in the pallium even exceeds those of primates^4^. In addition, connectivities of the ascending sensory pathways, associative forebrain areas, and subpallial structures have been suggested to be quite similar^36^. Hence, this neuronal complexity may explain why corvids attain feats equal to those of non-human primates. For instance, corvids have been suggested to be capable of skills such as recalling specific past events (episodic-like memory: scrub jays *Aphelocoma coerulescens*), planning for the future (common ravens *Corvus corax;* scrub jays), insightful problem-solving (New Caledonian crows *Corvus moneduloides;* rooks *Corvus frugilegus*), tactical deception (common ravens), and tool-use (New Caledonian crows*;* rooks)^40–45^. Common ravens, the most widely distributed member of the corvid family, are particularly renowned for their sophisticated social cognitive skills including the formation of coalitions, considering visual perspectives of others, and directing conspecifics’ attention to external referents^40, 46, 47^. Massen and colleagues^48^ thus speculated that ravens’ cognitive skills are primarily expressed in the social domain. But, recent studies also showed sophisticated physical cognitive skills such as mastering inference by exclusion, spatial memory, object permanence, and caching behaviour (for recent overviews see^40, 49^). The perceived difference between ravens’ physical and social cognitive skills may therefore be due to a lack of data only, rather than being the result of domain specificity (reviewed in^50^).

However, a comprehensive understanding of ravens’ (and other corvids’) cognitive abilities has been severely hampered by the use of single cognitive paradigms only^41, 51–55^. In addition, a systematic, quantitative comparison of corvids’ and non-human primates’ cognitive skills is non-existent. This is surprising, since a lot of research attention has been recently devoted to use or re-model the PCTB to enable cross-species and cross-taxa comparisons (e.g., monkeys^18^, parrots^56^, dogs^57^). Furthermore, research into the development of cognitive abilities of corvids and other non-human taxa is relatively scarce^58–60^. This is despite the fact that cognitive development stirred interest in the early days of comparative cognition^61, 62^, and forms (ontogeny) with causation (mechanism), survival value (fitness) and evolution (phylogeny) Tinbergen’s famous four why’s^63^.

To date, the existing studies on cognitive development in corvids have mainly been devoted to one of the most studied traditional sensorimotor skills, the object concept or object permanence (which entails the ability to represent objects that are out of view)^64^ and food-storing corvid species (but see^65^). European jays (*Garrulus glandarius*), magpies (*Pica pica*) and common ravens begin to store and retrieve their caches around the time of feeding independence and acquire sophisticated levels of object permanence (up to stage 6 in European jays and ravens) in their first year of life^66–68^. This rapid developmental phase stands in stark contrast to the much slower developmental pace in different species of psittacines^69,70^, which are also renowned for their sophisticated cognitive skills (e.g.,^49,71^). Similarly, a recent qualitative comparison of the development of Piagetian sensorimotor abilities across two bird and eleven mammal species (one corvid, one psittacine, five monkey, four great ape, and two carnivore species) showed that the developmental pace of ravens was markedly accelerated compared to that observed in the other species while the general developmental pattern was relatively similar^72^. This study, although only qualitative, marks a new trend in Cognitive Development since comparative research has traditionally been biased towards investigations of the cognitive development of human and non-human primates only^73,74^. For instance, Wobber and colleagues^11^ adapted the PCTB of Herrmann and colleagues^10^ to compare the development of cognitive skills between human children, bonobos, and chimpanzees. They found significant differences in the pattern and pace of cognitive development between the human and the two great ape model groups, with an accelerated ontogeny in children compared to individuals of the great ape species. In addition, divergent patterns of cognitive development were particularly apparent in the social domain, including for instance greater inter-relationships of social cognitive skills in children relative to apes (see also^75^).

Hence, to enable a more detailed understanding of cognitive performance across development in corvids and to address these critical gaps in our knowledge, we carried out a large-scale assessment of ravens’ cognitive skills across nine physical and six social cognitive tasks with a special focus on development. In addition, we revisited the claim that corvids rival non-human primates in their cognitive abilities^34,40^ by carrying out the first systematic, quantitative comparison of physical and social cognitive performance between ravens and individuals of two great ape species^76^. To do so, we applied the methodology of the PCTB^10^ as close as possible for a species using her beak instead of extremities (see also for adaption of size of material^18^).

The Corvid Cognition Test Battery (CCTB) was administered to eight hand-raised birds. The physical tasks comprised different cognitive scales involving spatial (investigating for instance spatial memory, and object permanence), quantitative (testing the ability to understand relative numbers and the addition of numbers), and causal tasks (examining causal reasoning via distinct cues such as sound and shape). The social tasks involved cognitive scales of social learning (for instance using information provided by the experimenter to solve a task), communication (for example taking into consideration the attentional state of a human experimenter) and theory of mind (for instance being able to understand the intentions of the experimenter) (for more details see Table 1, and the supplementary material). Also note that we adopted the original terms by Herrmann and colleagues^10^ to enable comparison between tasks and species. However, some tasks represent precursors to distinct skills only rather than full-blown cognitive abilities, for instance gaze following does not equal theory of mind. The CCTB was carried out during four distinct equally distributed time points after the birds had fully hatched: Four months of age, eight months of age, twelve months of age, and 16 months of age. The following detailed descriptions of the tasks have been adopted from the studies of Schmitt and colleagues^18^ and Herrmann and colleagues^10^. Concerning the number of trials per task and item, we followed the methodology of Schmitt and colleagues^18^.

**Table 1 Overview of the Corvid Cognition Test Battery:** Table 1 provides an overview of the Corvid Cognition Test Battery as a function of domain, scale, task, item, number of trials, chance probability, and description of scale.

Domain	Scale	Task	Item	No of trials	Chance probability	Description of scale
**Physical**	**Causality**	Noise		**12**		Causal understanding including tool use.
			Noise full	6	0.5	
			Noise empty	6	0.5	
		Shape		**12**		
			Board	6	0.5	
			Cloth	6	0.5	
		Tool Properties		**24**		
			Side	6	0.5	
			Bridge	6	0.5	
			Ripped	6	0.5	
			Broken wool	6	0.5	
	**Quantity**	Relative Numbers		**13**	0.5	Discriminating quantities.
		Addition Numbers		**7**	0.5	
	**Space**	Spatial Memory		**6**	0.33	Locating or tracking a rewards after location changes.
		Object Permanence		**18**		
			Single displacement	6	0.33	
			Double adjacent displacement	6	0.5	
			Double non-adjacent displacement	6	0.33	
		Rotation		**18**		
			180° middle	6	0.33	
			360°	6	0.33	
			180° side	6	0.33	
		Transposition		**18**		
			Single transposition	6	0.33	
			Double baited transposition	6	0.33	
			Double unbaited transposition	6	0.33	
**Social**	**Communication**	Comprehension		**18**		Understanding and producing communicative signals.
			Look	6	0.5	
			Point	6	0.5	
			Marker	6	0.5	
		Pointing Cups		**8**	0.5	
		Attentional State		**4**		
			Away	1	Unknown	
			Towards	1	Unknown	
			Away Body facing	1	Unknown	
			Towards Body-away	1	Unknown	
	**Social learning**	Social Learning		**3**		Solving a simple but not obvious problem by observing a demonstrated solution.
			Apparatus 1	1	Unknown	
			Apparatus 2	1	Unknown	
			Apparatus 3	1	Unknown	
	**Theory of Mind**	Gaze Following		**9**		Following the gaze of an actor.
			Head and Eyes	3	Unknown	
			Back	3	Unknown	
			Eyes	3	Unknown	
		Intentions		**12**		Understanding what an actor intended to do.
			Trying	6	0.5	
			Reaching	6	0.5	

Numbers in bold depict the number of trials carried out per task.

Note: Some parts of the table are taken from Herrmann and colleagues^4^*.*

We addressed the following three research questions:

1. Do ravens perform differently in the domains of physical and social cognition?
  To investigate this question, we compared the performance of the ravens in the physical cognitive tasks to their performance in the social cognitive tasks. Based on previous findings^48^, and given that ravens live in complex social systems consisting of fission–fusion dynamics and long-term monogamy^40,47^, we predicted to find higher scores in the social than in the physical cognitive domain.
2. How does cognitive performance develop in ravens?
  To address this question, we compared the performance of all individuals across four different time points: four months of age, eight months of age, twelve months of age, and 16 months of age. Based on the existing studies of cognitive development in ravens^68,72,77^, we predicted to find a relatively rapid development across cognitive scales and the four investigated time points.
3. Do ravens match great apes’ cognitive abilities?
  To investigate this question, we quantitatively compared the cognitive performance of the ravens in the CCTB to the cognitive performance of chimpanzees and orang-utans in the PCTB^10^. Since ravens are known to exhibit a variety of socio-cognitive traits necessary to manoeuvre successfully through their complex social world^34,48^ and have been suggested to be social rather than physical intellects (but see for tool performance and physical cognitive skills^42,48^), we predicted to find species differences between ravens and great apes in the physical cognitive domain only.

## Methods

### Birds and study site (see for methods, birds and apparatuses used^76^)

All birds had been taken from their captive parents at the age of three weeks (April/May 2014) and had been hand-raised in the corvid aviaries of the Max-Planck Institute for Ornithology in Seewiesen, Germany. The first weeks (until the end of May 2014), the ravens were hand-reared in artificial nests (chicks originating from the same parents were kept in the same carton box with wooden sticks and leaves). This took place in a smaller room to mimic “natural” conditions as good as possible. Only after fledging (~ 45 days, end of May 2014), the birds were moved to the outdoor aviary. The group of ravens consisted of four sibling pairs, which were marked with coloured rings on their legs for identification. Immediately after fledging, all our birds were trained using positive reinforcement techniques (rewarding the animal when it performs the target behaviour, waiting at their starting perch, etc.) to be able to be individually separated within the test compartments. Prior to the start of the CCTB, all birds were familiarised with the experimental equipment (e.g., wooden boxes with holes, plastic bottles, etc.), and the cameras. The test participation was always voluntary. If a bird did not engage during testing (e.g., did not make a choice), it was released to the group and tested on the subsequent day. Hence, none of our individuals had prior testing experience other than habituation to the test facilities and training to interact with the human experimenters. Since one bird stopped participating voluntarily in the second experimental set, we did not continue testing this individual for the rest of the experiment (see Table 2). Testing took place from Monday to Friday (sometimes Saturday) between 08:00–12:00 a.m. and between 02:00–04:00 p.m.

**Table 2 The ravens:** Table 2 provides information about the tested birds (name, sex, and sibling group named after their origin).

Individual	Sex	Sibling Group (Origin)
Arthus	Male	Zoo Altenfelden
Aramis	Female	Zoo Altenfelden
Maxi	Female	Wild Park Bayrischer Wald
Moritz	Male	Wild Park Bayrischer Wald
Rhea	Female	Vogelpark Walsrode
Munin	Female	Vogelpark Walsrode
Bonny	Female	Zoo Wels
Clyde	Male^1^	Zoo Wels

^1^Individual stopped participating during the second set of experiments and was not tested further.

**Figure 1 The raven aviaries:**
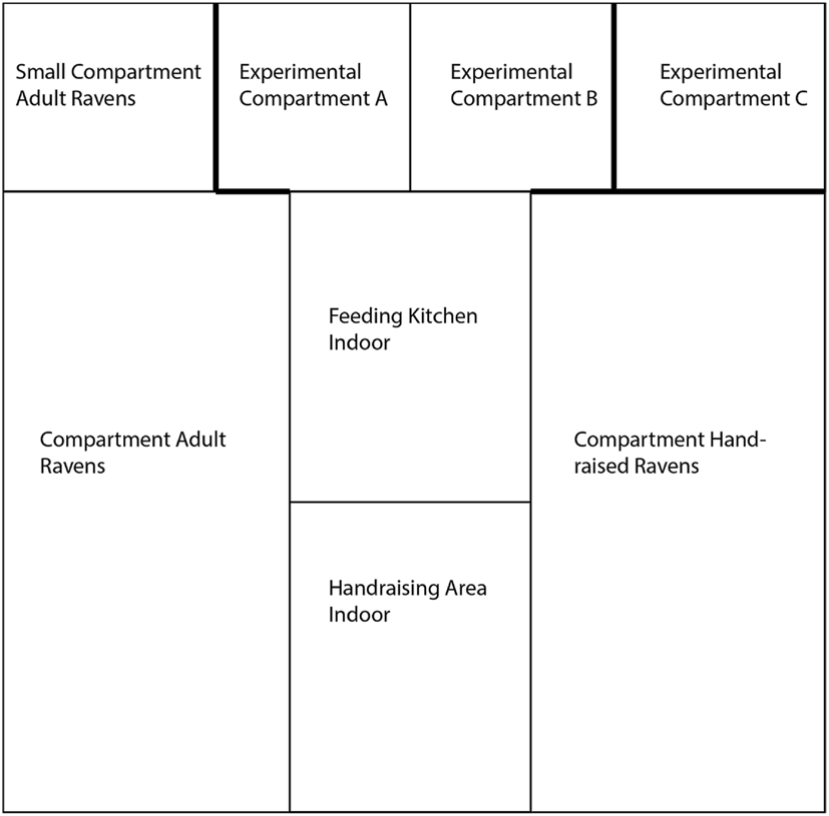
Figure 1 depicts a sketch of the raven aviaries in Seewiesen. The thick lines represent opaque site elements/fences.

The raven aviaries (see Fig. 1) were composed of one big (12 × 4.3 × 5.3 m) and three small sections (one section: 3.8 × 2.9 × 2.9 m, and two sections: 2.14 × 2.9 × 2.9 m), and all contained natural vegetation (e.g., perches) and diverse ground cover including soil and gravel. The ravens were fed twice a day between 07:00–08:00 a.m. and 04:00–05:00 p.m. with various types of meat, dairy products, mealworms and fruits. Water was freely available throughout the day. In the first experimental set, when the birds were not yet flying/moving around a lot, we videotaped all experiments with one video camera (Canon Legria HF S10). In the other three experimental sets, we used two cameras (Canon Legria HF S10 and Canon HF M41). We placed the cameras two meters away from the testing compartment to avoid disturbing the birds. One of the cameras was placed in Experimental Compartment A behind the experimenter, and the second camera was placed in the Feeding Kitchen to enable filming through the window (see Fig. 1).

### Testing apparatus, general procedure and habituation

The testing apparatus was located in the same compartment as the experimenter and the bird had to indicate her choice by pointing/touching through the wire mesh (see Fig. 1). To keep the birds motivated, we used highly desired food rewards, which were only available in the experimental context (pieces of peanuts, pieces of dog treats “Frolic”, skin of porks “Grammeln”). The testing was done by two experimenters, MJS and CRB. They had hand raised the ravens with the help of volunteers, and were highly familiar with all birds since their arrival in Seewiesen. The birds were tested during four developmental time points, at four months (July/August 2014), eight months (November/December 2014), twelve months (March/April 2015), and 16 months (July/August 2015) of age. MJS was the main experimenter during the first two time points and experimental sets, whereas CRB was the main experimenter during the second two time points and experimental sets of testing (see Table 1 for a detailed description of the amount of trials and tasks).

### Experimental setup

**Figure 2 Experimental set-up:**
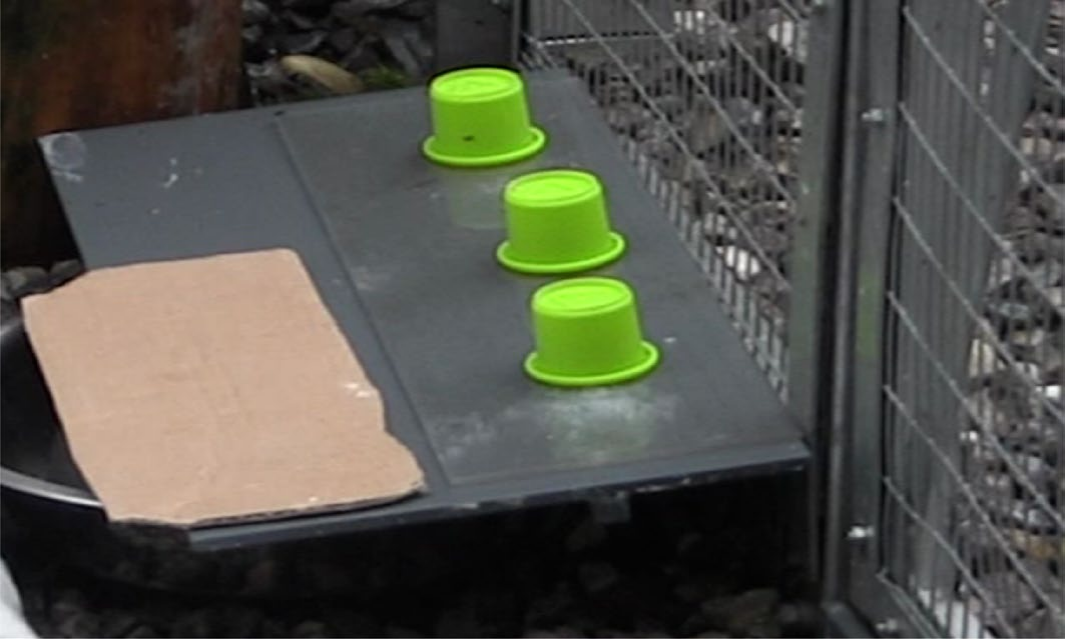
Figure 2 shows a picture of the experimental set-up with the experimental board located on two stone blocks, a transparent sliding board, and testing material (three green cups) in front of the bird’s  experimental compartment.

During the experiments, the birds were separated (physically and visually) from their group members (see Fig. 1; the tested bird was located in Experimental Compartment B, the rest of the group in the Compartment for Handraised Ravens). The human experimenter sat in a second compartment (see Fig. 1; Experimental Compartment A; again physically and visually isolated from the rest of the group) and interacted with the bird through the wire mesh that separated the two testing compartments. The testing apparatus used during the majority of the experiments (exception Social Learning, Gaze following and Pointing Cups) consisted of a grey polyvinylchloride board located on two stone blocks and a transparent sliding board also made of polyvinylchloride (see Fig. 2). The sliding board was lying on top of the grey board. Three cups were used to cover/present the food reward. These were placed on the sliding table.

### Physical domain

#### Scale:Space

- Spatial memory
  Three cups were placed in a row on the platform. The experimenter showed the bird two rewards, and placed them under two adjacent cups of the three cups in full view of the bird. Then the platform was pushed towards the bird, and it was allowed to make two choices in succession by pecking against the cups. If, however, the bird chose the empty cup first, it was not allowed to make further choices.
  The response was counted as correct when the bird had chosen both baited cups in succession.
- Object permanence
  Three cups were placed in a row on the platform. An additional small opaque cup was used. The experimenter baited this small cup while the bird was watching. The small cup was then moved towards one of the larger cups, which was slightly lifted by raising the side not facing the bird. The experimenter then made a swapping movement with the small cup, as if swapping the reward under the larger cup. The experimenter also touched the other cups to avoid local enhancement. After moving the small opaque cup under the specific larger cup, the experimenter lifted the small cup to show the bird that the small cup was now empty. The platform was pushed forward to allow the bird to choose.

There were three possible displacements performed:

*Single displacement*: The experimenter moved the small cup hiding the reward under one of the three cups, as described above, and swapped the reward under it.
*Double adjacent displacement*: The experimenter moved the small cup hiding the reward under two adjacent cups in succession, as described above, and left the reward under one of these cups.
*Double non-adjacent displacement*: The experimenter moved the small cup hiding the reward under the left and right cup in succession, as described above, and left the reward under one of them.

A correct response was counted when the bird had chosen the baited cup.

c. Rotation

Three cups were placed in a row on a cardboard, which was then placed on the platform. The experimenter showed a reward to the bird, and placed it under one of the three cups while the bird was watching. Then the tray was rotated in three possible ways:

*180° middle*: The reward was placed under the middle cup, and the tray was rotated 180° in clockwise or counter clockwise direction (counterbalanced). After the rotation, the reward was located at the same position as it was initially placed.
*360°*: The reward was placed under either the left or right cup, and the tray was rotated 360° in clockwise or counter clockwise direction (counterbalanced). After the rotation, the reward was located at the same position as it was initially placed.
*180° side*: The reward was placed under either the left or right cup (counterbalanced), and the tray was rotated 180° in clockwise or counter clockwise direction (counterbalanced). After the rotation, the reward was located on the opposite side of where it was initially placed.

After the completed rotation, the bird was allowed to choose one cup. A correct response was scored when the bird chose the baited cup first.

d. Transposition

Three cups were placed in a row on the platform in front of the experimental compartment. The experimenter showed a reward to the bird, and afterwards placed the reward under one of the three cups while the bird was watching. Then one of three possible manipulations was performed:

*Single transposition*: The experimenter switched the position of the baited cup with one of the empty cups. The third cup was not touched.
*Double unbaited transposition*: The experimenter switched the position of the baited cup with one of the empty cups. Then the positions of the two empty cups were switched.
*Double baited transposition*: The experimenter switched the position of the baited cup with one of the empty cups. Then the position of the baited cup was switched again with one of the empty cups. After the transpositions were completed, the bird was allowed to choose one cup.

A correct response was scored if the bird chose the baited cup first.

#### Scale: quantities

- Relative Numbers

The experimenter placed two small rectangular cardboard pieces (10 × 10 cm) on the platform and lifted an occluder to prevent the bird from watching the baiting procedure. Then the experimenter baited the cardboard pieces with different amounts of equally sized food pieces (1/8 of a Frolic piece). The experimenter then placed the cardboard pieces in the middle on the platform, and removed the occluder so that the bird could see the amounts lying on each board. After ~ 5 s had passed and the bird had paid attention, the experimenter moved the plates simultaneously to the sides of the platform, one to the right and one to the left. The sliding table was pushed to the front, and the bird was allowed to choose and obtain all food pieces lying on the respective plate. Each bird received one trial for each of the following pairs of numbers (the order was randomized but constant among birds): 1:0, 1:2, 1:3, 1:4, 1:5, 2:3, 2:4, 2:5, 2:6, 3:4, 3:5, 3:6, 3:7, 4:6, 4:7, 4:8 (the side was counterbalanced).

A correct response was scored if the bird chose the larger quantity first.

b. Addition Numbers

The experimenter placed two small rectangular cardboard pieces on the platform, and lifted an occluder to prevent the bird from watching the baiting procedure. Then the experimenter baited the two cardboard pieces with different amounts of reward (same as in Relative Numbers). She/he also baited a third cardboard piece, which was placed in the middle. Then the three boards were covered with cups and placed in the middle of the platform. After the occluder was removed, the experimenter lifted the cups of the two outer cardboards simultaneously. After ~ 5 s had passed, the experimenter covered the two outer plates again and uncovered the cardboard in the middle. The bird was able to view the amount lying on the middle cardboard for ~ 5 s. Then the experimenter transferred the rewards from the middle plate to one of the side cardboards. During the transfer, the bird could not see the content of the side cardboard boards because they were still covered with the cups. Then the experimenter removed the empty cardboard in the middle, and the bird was allowed to choose between the two covered cardboards on the outer sides (the order was randomized but constant among bird): 1:0 + 3:0 = 4:0; 6:1 + 0:2 = 6:3, 2:1 + 2:0 = 4:1, 4:3 + 2:0 = 6:3, 4:0 + 0:1 = 4:1, 2:1 + 0:2 = 2:3, 4:3 + 0:2 = 4:5 (the side was counterbalanced).

A correct response was scored if the bird chose the larger quantity first.

#### Scale: Causality

- Noise

The experimenter placed two cups on the platform, and lifted an occluder to prevent the bird from observing the baiting. Then the experimenter put a reward (peanut) in one of the two cups, and closed both cups with the small cardboard board already used in the Quantity task. After the occluder was removed, one of two possible manipulations were performed:

*Noise full*: The experimenter shook the baited cup three times, so that the food rattled inside, and only lifted the empty cup without shaking it. Whether the experimenter started with the baited or empty cup was randomized.
*Noise empty*: The experimenter shook the empty cup (which produced no sound) three times, and then lifted the baited cup without shaking it. Whether the experimenter started with the baited or empty cup was randomized.

After the manipulations, the bird was allowed to choose one cup. A correct response was scored if the bird chose the baited cup first.

b. Shape

The experimenter placed an occluder and placed two identical items (see items below) on the platform. The experimenter showed the bird the reward (1/8 of a Frolic), and placed it underneath one of the two identical objects causing a visible inclination or bump. After this procedure, the occluder was removed, and the bird was allowed to make a choice.

*Board*: The experimenter hid the reward underneath one of two cardboard pieces (10 × 10 cm). The reward caused a visually apparent inclination as it was placed on the food (the other board remained flat on the table).
*Cloth*: The experimenter hid the reward underneath one of two pieces of white cloth (4 × 2 cm). The reward made a visible bump under the piece of cloth where it had been hidden (the other cloth remained flat on the table).

A correct response was scored if the bird chose the baited board or baited cloth first.

c. Tool properties

The experimenter lifted an occluder and placed two different tools on the platform. One tool was functional and could be used to retrieve a reward associated with it (e.g., lying on top of it). In contrast, the second tool was non-functional, and could not be used to obtain the reward. The following manipulations were conducted:

*Side*: The experimenter put two identical pieces of white cloth (4 × 2 cm) on the platform behind an occluder, and placed a reward on top of one cloth piece. The other reward was placed directly next to the other piece of cloth (i.e., making the second tool ineffective for retrieving the food). After the occluder was removed, the bird had to choose the functional tool by either pulling the piece of cloth with the reward on top of it, or by pecking against the functional piece of cloth.
*Bridge*: The experimenter put two identical small plexiglass bridges over each of the far ends of the two identical pieces of cloth behind an occluder. One reward was then placed on top of the bridge (making the tool ineffective in retrieving the food). The other reward was placed on the cloth underneath the bridge. After removing the occluder, the bird had to choose the functional tool by either pulling the cloth with the reward placed directly on it, or by pecking against the functional piece of cloth.
*Ripped*: The experimenter put up an occluder and placed a rectangular, intact piec of cloth on one side of the table and two smaller cloth pieces on the other side. She/he arranged the small pieces of cloth in a way that there was a 1 cm gap between them. Then one reward was placed on top of the far end of the intact cloth. The other reward was placed on the out of reach piece of the two disconnected pieces (making the tool ineffective to retrieve the reward). After removing the occluder, the bird had to choose the functional tool by either pulling the cloth with the reward placed directly on it, or by pecking against the functional piece of cloth.
*Broken wool*: The experimenter put up an occluder, and placed two strings of wool on the platform. One string was cut into two pieces. Similarly to the *Ripped * condition (see above) both strings were arranged in a way that the gap was visible, but that both pieces showed equal length. A peanut was tied to the far end of the wool strings out of the bird’s reach. After removing the occluder, the reward could only be retrieved by pulling the intact piece of wool.

A correct response was scored if the bird first chose the functional tool by pulling it or by pecking against it.

### Social domain

#### Cognitive scale: Social learning

First, the bird had two minutes to solve the apparatus without demonstration by a human experimenter. If the bird did not succeed, the test condition started. The experimenter demonstrated the solution and handed the reward to the bird. Afterwards, the bird had again two minutes to solve the problem. To count as a correct response, the bird had not only to obtain the reward but do so by using a similar procedure as the one demonstrated by the experimenter. We used different apparatuses (see for further details supplementary material) during the different testing periods, to rule out performance based on generalisation and experience with the tasks.

#### Cognitive scale: Communication

- Comprehension

The experimenter placed two cups on the testing platform behind an occluder, one on the left and the other one on the right side. The experimenter showed the bird the reward, and let the reward then disappear behind the occluder. Subsequently, the experimenter hid a reward under one of the cups, removed the occluder, and gave one of the three following social cues:

*Look:* The experimenter sat behind the platform and alternated her/his gaze between the bird and the baited cup while calling the bird’s name. After these gaze alternations, she/he continuously looked towards the cup.
*Point*: The experimenter sat behind the platform and continuously pointed to the baited cup using the extended index finger of her/his cross-lateral hand. At the beginning of the pointing, the experimenter alternated her/his gaze between the bird and the cup three times and called the bird’s name. Subsequently, she/he continously looked towards the cup.
*Marker*: The experimenter held an iconic photo marker, which depicted the reward in her/his hand, and alternated the gaze three times between the photo marker and the bird while calling the bird’s name. Then the experimenter placed the photo on top of the baited cup. On the other cup, the experimenter placed an empty piece of paper, which had the same size. Both pictures were placed at the same time.

After providing one of these cues, the bird was allowed to choose one cup. A correct response was scored if the bird chose the baited cup first.

b. Production: pointing cups

Two cups served as hiding places for a food reward. These cups were placed in a distance of two meters to each other and close to the fence of the experimental compartment. The cups did, however, not touch the fence. Hence, the bird was not able to touch the cups with its beak. The second experimenter (E2) entered the testing area, placed a reward under one of the two cups while the bird was watching, and then left the testing area. Then the first experimenter (E1) entered the testing area and sat down equidistant to the two cups. She/he waited until the bird approached one cup and pointed towards it with its beak through the wire mesh.

A correct response was scored, if the bird chose the correct cup first within one minute.

c. Production: attentional state

E2 entered the testing area and placed a reward out of reach but in front of the birds’ experimental compartment on the bird’s right or left side. Then E2 left the area and E1 entered the experimental compartment. She/he stood on the end of the room opposite of the reward and pretended not to see the reward on the floor. E1 stood and the four following behaviours:

*Away*: E1 turned around and looked away from the reward. When the bird approached E1 and walked into her/his visual field within 20 s, E1 turned around and waited whether the bird directed her/his attention to the reward. If the bird went back to the reward’s location and indicated the reward within 20 s, E1 handed the reward to the bird.
*Towards*: E1 looked towards the reward. When the bird approached the reward and directed E1′s attention towards the reward within 20 s, E1 handed it over to the bird.
*Away Body-facing*: Identical to *Away*, except that E1’s body faced toward the reward while the face was turned away. When the bird approached E1, walked in her/his visual field and directed the experimenters attention towards the reward within 20 s, E1 handed it over to the bird.
*Towards Body-away*: Identical to the *Towards* condition, with the exception that the body of E1 was turned away and the face was directed towards the reward. When the bird approached the reward and directed E1′s attention towards the reward within 20 s, E1 handed it over to the bird.

#### Cognitive scale: Theory of mind

- Gaze Following

*Baseline:* As baseline condition, the experimenter sat for two minutes in front of the experimental compartment and looked at the subject. All look-ups from the bird were counted to calculate a baseline level (look-ups per min).

In the experimental condition, the experimenter sat in front of the bird and handed a piece of food to the bird to attract the bird’s attention. When the bird came closer and looked at the experimenter, the trial started. The gaze cue was conducted in three different ways:

*Head* + *Eyes*: The experimenter called the bird’s name and showed a piece of food. Then the experimenter hid the food in her/his hand, which remained in front of her/his body. Afterwards the experimenter looked up for ~ 10 s by lifting up the head and the eyes.

*Back*: The experimenter sat with her/his back facing the bird. The experimenter called the bird’s name and showed a piece of food to the bird. Then the food was hidden in the experimenter’s hand, which remained in front of the experimenter’s body. Afterwards the experimenter looked up in the air for ~ 10 s.

*Eyes*: The experimenter called the bird’s name and showed the bird a piece of food. Then the experimenter hid the food in her/his hand, which remained in front of the experimenter’s body. Afterwards, the experimenter glanced up in the air for ~ 10 s without moving the head, meaning her/his face was still facing the bird as before.

A correct response was scored if the bird followed the gaze of the experimenter (movement of the head to face upwards or tilting of the head resulting in one eye gazing upwards).

b. Intentions

E1 put an occluder on the platform and placed two cups. She/he showed the reward to the bird, and then hid it in one of the two cups. After removing the occluder, E2 manipulated the cups in one of the two following ways:

*Trying:* E2 reached for the baited cup and tried unsuccessfully to remove the lid while looking at the cup.

*Reaching:* A plexiglass barrier blocked E2′s access to the two cups. She/he unsuccessfully tried to gain access to the baited cup by extending the equilateral arm and simultaneously looking at the correct cup. E2 continued to give this cue until the bird made a choice.

After each demonstration, E1 approached the table after ~ 3 s and pushed the platform forward so that the bird was allowed to make a choice. To count as a correct response, the bird had to choose the baited cup first.

All trials were done in order, categorical by task, and using the same order as applied in the PCTB^10^.

### Scoring and reliability

Great apes use their hands to explore objects, while ravens manipulate objects with their beaks and feet. Thus, in contrast to the procedure of Herrmann and colleagues^10^, a choice was scored when the tested individual pointed with the beak through the wire mesh at one of the locations of the objects (cups or other material), or pecked against the cup/material. When the tested bird pointed at the correct location, it was given the opportunity to retrieve a small food reward. When it made incorrect responses (except otherwise stated), the experimenter showed the location of the hidden food after each trial, took the food away and did not give any reward to the bird. Scoring took place by both experimenters during testing (in all tasks except gaze following). In the gaze following task, a second observer coded the videotapes to assess inter-observer reliability, resulting in an ‘excellent’ level of agreement (Cohen’s K = 0.93).

### Statistical analyses

To investigate how the proportions of correct responses of ravens varied with age and cognitive scale, we used a Generalized Linear Mixed Model (GLMM^78,79^) with a logit link function. The response in this model consisted of the proportion of correct trials. In R such an analysis of proportions of binary outcomes is possible with the response being a two columns matrix consisting of the number of successes and failures per trial respectively^80^. As predictors with fixed effects, we included age and scale as our key test predictors, and sex and experimenter (two levels) as control fixed effects. Because we predicted a scale dependent change of the performance throughout ontogeny, we incorporated the two-way interaction between age and scale as another test predictor with fixed effect. As random effects, we included the identity of the bird and the sibling group, as well as the item and the task and also the trial ID into the model. To control for varying chance probabilities across the cognitive tests, we included chance probability (log-transformed) of the different items as an offset term into the model^79^.

To keep type I error rate at the nominal level of 0.05^81,82^, we included all theoretically identifiable random slopes components (age, scale, experimenter, and their interaction within bird identity and sibling group; sex within sibling group; age, sex, and experimenter within item and scale; we manually dummy coded and then centred factors before entering them into the random slopes part of the model). Initially, we also incorporated all correlations between random intercepts and slopes. However, most of them appeared to be unidentifiable, as indicated by absolute correlation parameters being essentially one^83^. Hence, we removed them from the model.

Since chance probabilities for the items in the tasks social learning, attentional state and gaze following cannot be determined (see Table 1), we excluded these from the model. To assess the overall effect of our key test predictors, we compared the fit of the full model (with interaction, fixed factors and random effects) with that of a null model^82^ comprising only the control fixed effects predictors, the random effects, and the offset term using a likelihood ratio test^84^.

To assess model stability, we compared the estimates obtained from the model based on all data with those obtained from models with the levels of the random effects excluded one at a time. The results showed that the model was relatively unstable with regard to the effect of the two-way interaction.

Overdispersion appeared to be no issue (dispersion parameter: 1.00). To rule out collinearity, we assessed Variance Inflation factors (VIF^85^) for a standard linear model excluding the interaction, the random effects, and the offset term. With maximum VIF of 3.86 for age and 3.84 (squared Generalized VIF taken to the power of 1/2 × the respective degrees of freedom^86^) for experimenter collinearity was not severe.

We fitted the model in R (version 3.4.0^87^) using the function glmer of the R package lme4 (version 1.1–13^88^). Confidence intervals were obtained using the function bootMer of the package lme4, using 1000 parametric bootstraps and bootstrapping over the random effects, too (argument ‘use.u’ set to FALSE). We derived tests of the individual fixed effects using likelihood ratio tests comparing the fit of the full model with that of models lacking the terms to be tested one at a time (^81^*; R function drop1 with argument ’test’ set to "Chisq"*). Prior to fitting the model, we z-transformed age to a mean of zero and a standard deviation of one. The sample size for this model was 754 tests of eight ravens in four sibling groups, tested in twelve tasks and with 26 items.

To compare performance levels among species, we also used a GLMM with logistic error structure and logit link function. The response was again a matrix with the numbers of correct and incorrect responses. Into the model, we included as key test predictors with fixed effects species and its interaction with scale. To control for sex effects, we included sex as an additional fixed effect (and also the main effect of scale). As random intercepts effects, we included item, task, individual, task nested in individual, and trial ID. As random slopes, we included species and sex within item and task and scale within individual (all manually dummy coded and then centred). As for the first model, we included chance probability (log-transformed; see Table 1) of the different items as an offset term into the model. The null model lacked species and its interaction with scale but was otherwise identical to the full model. The model was not overdispersed (dispersion parameter: 0.881), and collinearity was no issue either (maximum squared Generalized VIF: 1.02). We determined model stability and confidence intervals as for the first model. The sample size was a total of 4342 trials, conducted with eight individuals using 26 items and twelve tasks (with 1752 task nested in individual).

Finally, we fitted an additional model for species comparison, using only those items for which the probability of a correct response was unknown (see Table 1). This model was identical to the other species comparison model, with the exceptions that it did not include an offset term, lacked the random effects of task and task nested in individual, and that the fixed effect of scale comprised only the levels Causality, Communication, Quantities, Space, and Theory of Mind. The model was somewhat underdispersed (dispersion parameter: 0.524). The sample size for this model was a total of 1611 trials conducted with eight individuals using eight items.

### Ethical note

All applicable national, and institutional guidelines for the care and use of animals were followed. In accordance with the German Animal Welfare Act of 25th May 1998, Section V, Article 7, the study was classified as non-animal experiment and did not require any approval from a relevant body.

## Results

**Figure 3 Correct responses as a function of cognitive scale:**
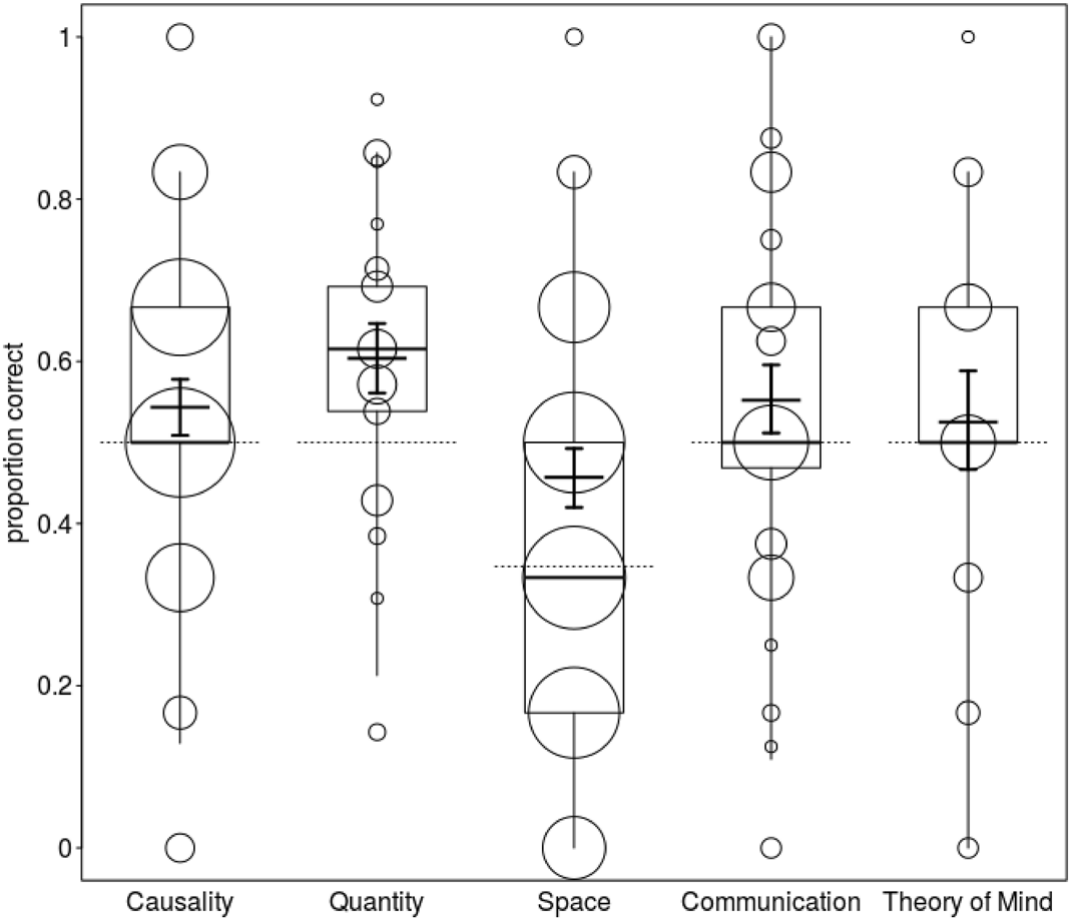
The figure shows the proportions of correct responses as a function of cognitive scale. Dots represent the amount of observations per proportion correct (N = 1 to 88 tests). Indicated are median (horizontal lines), quartiles (boxes), and percentiles (2.5 and 97.5%, vertical lines). The crosses represent the fitted model and its confidence limits (conditional on all covariates and factors centred to a mean of zero). The doted horizontal lines depict chance probability.

To test whether ravens’ cognitive performance differed in relation to social or physical cognitive tasks (question 1), we fitted a model with an interaction between scale and age. Overall, the full-null model comparison was significant (likelihood ratio test: chi-square = 17.265, df = 9, *P* = 0.045), but the interaction between age and scale did not reveal significance (chi-square = 2.417, df = 4, *P* = 0.660; see Table S1 and S2 in the supplementary material). After removal of the non-significant interaction, we found that the performance of the birds was on average significantly higher in quantitative skills as compared to all others. In addition, spatial skills were significantly lower as compared to all others (see Fig. 3; for further details see Table S3 and S4 in the supplementary material).

**Figure 4 Proportion of correct responses as a function of ravens’ age:**
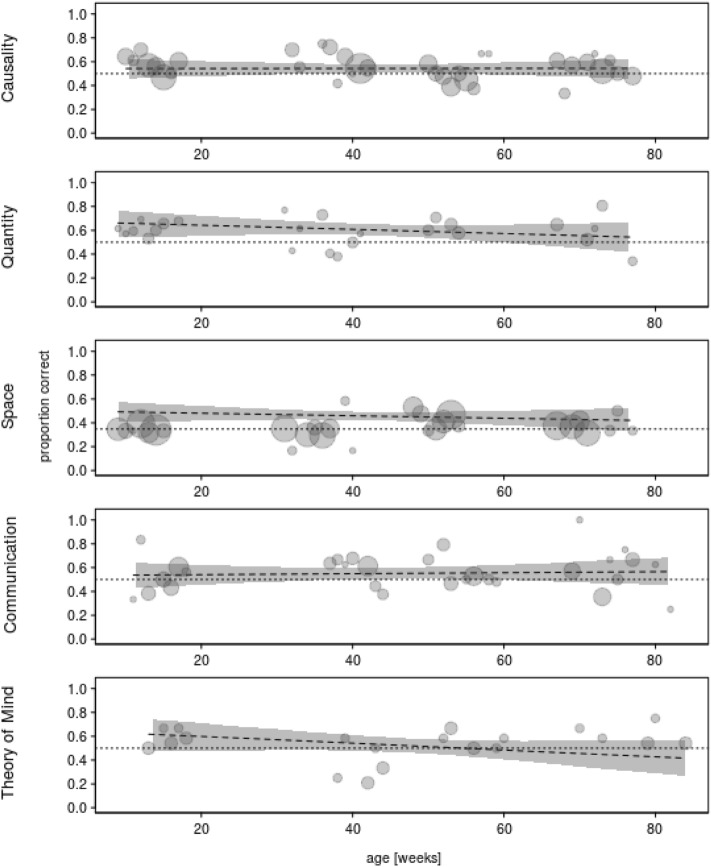
The figure depicts the proportions of correct tests per week of age (in weeks). The area of the dots represents the number of tests per week (N = 1 to 24 tests). The dashed line and grey area represent the fitted model and its confidence interval based on all covariates and factors, except age centered to a mean of zero. The doted horizontal lines depict chance probability.

The same model was used to investigate the ontogeny of cognitive skills in ravens (question 2). Their performance did not vary strongly over the course of the study period (− 0.063 ± 0.062, chi-square = 1.005, df = 1,* P* = 0.316, see Fig. 4).

With regard to the random effects, we found that these were mostly estimated to contribute very little to the probability of a correct choice. The clearly strongest random effects were the random slopes of experimenter within item, task, individual, and sibling group. Furthermore those of the interaction between age and scale space within individual and of scale communication within individual (see Table S2). The latter suggest that individuals varied in parts considerably with regard to how their scale specific performance varied with age.

**Figure 5 Proportions of correct responses as a function of species:**
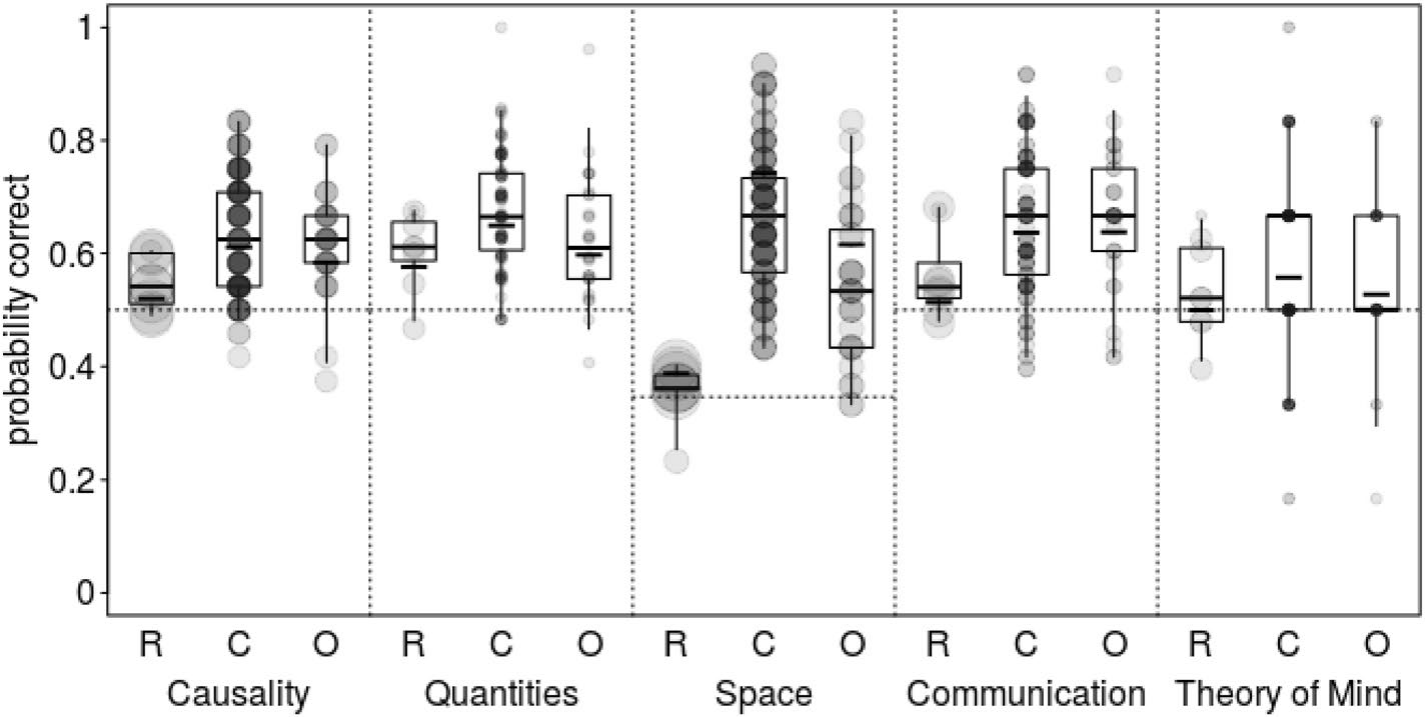
The figure shows the proportions of correct responses as a function of species (R: ravens; C: chimpanzees; O: orang-utans) and cognitive scale. The area of the dots represents the number of tests per individual and proportion correct (N = 2 to 40); darker dots represent tied observations. Indicated are median (horizontal lines), quartiles (boxes), and percentiles (2.5 and 97.5%, vertical lines). The short thick horizontal lines depict the fitted model. The doted horizontal lines depict chance probability.

To examine whether ravens rival great apes in cognitive skills (question 3), we fitted a model with an interaction between species and scale. Into this model, we only included those tasks for which the probability of a correct response was known (all physical cognitive tasks [scales: Causality, Spatial, Quantity] and the social cognitive tasks comprehension, pointing cups (scale: Communication), and intentions (scale Theory of Mind; see Table 1 and Table 3). The model revealed clear species differences (full-null model comparison: chi-square = 32.123, df = 10, *P* < 0.001). Furthermore, the full model showed a significant interaction between species and scale (chi-square = 15.008, df = 8, *P* = 0.059). Ravens showed a lower performance than the two great ape species in spatial skills. The performance of ravens and great apes in quantitative and theory of mind skills was similar. Concerning causal and communicative skills, it was slightly below that of great apes (see Table 3, Fig. 5, and Table S5 and S7 in the SA for details; see also S8 for raw data comparison).

**Table 3 Addressing species difference in performance:** The table depicts the results of the full model addressing species difference in performance as a function of Scale (estimates, together with standard errors and confidence intervals as well as minimum and maximum of estimates obtained when excluding levels of random effects one at a time).

Term	Estimate	SE	Lower Cl	Upper Cl	Min	Max
Intercept	0.875	0.224	− 0.345	0.516	0.823	0.927
Species Chimpanzee^(1)^	0.374	0.255	− 0.104	0.906	0.317	0.432
Species Orangutan^(1)^	0.259	0.217	− 0.154	0.691	0.197	0.315
Scale Communication^(2)^	− 0.025	0.355	− 0.705	0.678	− 0.098	0.103
Scale Quantities^(2)^	0.227	0.411	− 0.558	1.071	0.137	0.312
Scale Space^(2)^	− 0.533	0.321	− 1.173	0.054	− 0.605	− 0.380
Scale ToM^(2)^	− 0.080	0.447	− 0.977	0.832	− 0.220	0.060
Sex male^(3)^	0.049	0.043	− 0.038	0.131	0.030	0.066
Species Chimpanzee: Scale Communication	0.133	0.401	− 0.650	0.896	− 0.057	0.429
Species Orangutan: Scale Communication	0.253	0.327	− 0.422	0.853	0.096	0.474
Species Chimpanzee: Scale Quantities	− 0.064	0.421	− 0.852	0.744	− 0.207	0.085
Species Orangutan: Scale Quantities	− 0.170	0.319	− 0.850	0.433	− 0.215	− 0.109
Species Chimpanzee:Scale Space	1.139	0.369	0.421	1.841	0.760	1.462
Species Orangutan:Scale Space	0.666	0.317	0.024	1.295	0.319	0.829
Species Chimpanzee:Scale ToM	− 0.142	0.497	− 1.029	0.886	− 0.232	− 0.046
Species Orangutan:Scale ToM	− 0.149	0.406	− 0.944	0.655	− 0.225	− 0.074

^(1)^ Dummy coded, the reference category was Raven.

^(2)^ Dummy coded; the reference category was Causality.

^(3)^ Dummy coded with female being the reference category.

With regard to the random effects in the species comparison model, we found that some of the random slopes of scale within individual and of species within task were estimated to contribute considerably to the response (see Table S5). This suggests that the effects of scale were in part differing considerably between individuals and that species differences were in part varying considerably between tasks. Furthermore, we also found the estimated random intercept effects of individual and item to be quite large. This implies that the probability of solving a given problem varied quite considerably between individuals and items.

The model using only those tasks for which chance probability was unknown did not reveal a significant species difference (chi-square = 7.914, df = 6, *P* = 0.244). This means that the performance of our ravens and the great ape individuals did not differ considerably in social learning skills, communicative skills (Attentional State task), and theory of mind skills (Gaze following task; see Table 4 and Table S6 in the SA). Some of the random effects were estimated to contribute considerably, implying that the probability to solve a given problem varied in part strongly among individuals and items (see Table S6).

**Table 4 Species comparison for behaviours with unknown chance probability:** Table 4 depicts species comparison for behaviours with unknown chance probability.

Term	Estimate	SE
Intercept	− 0.216	0.618
Species Chimpanzee^(1)^	− 0.797	0.662
Species Orangutan^(1)^	− 1.147	0.487
Scale Social Learning^(2)^	− 3.576	1.246
Scale Theory of Mind^(2, 4)^	− 1.185	0.920
Sex Male^(3)^	− 0.039	0.179
Species Chimpanzee: Scale Social Learning	1.748	1.331
Species Orangutan: Scale Social Learning	1.742	0.804
Species Chimpanzee: Scale Theory of Mind^(4)^	0.263	0.986
Species Orangutan: Scale Theory of Mind^(4)^	0.397	0.692

^(1)^Dummy coded, the reference category was Raven.

^(2)^Dummy coded, the reference category was scale Communication only including the task Attentional State.

^(3)^Dummy coded with female being the reference category.

^(4)^Only including the task Gaze Following.

## Discussion

Here, we provide the first quantitative, large-scale investigation of physical and social cognitive skills in a large-brained songbird species—ravens. We particularly examined the effect of development on cognitive performance, and revisited the claim that corvids rival non-human primates in their cognitive abilities^34,40^. To achieve these goals, we fine-tuned one of the most elaborate large-scale cognitive test batteries—the PCTB^10^—to raven features. The results demonstrated that our ravens showed comparable cognitive performance in the domains of social and physical cognition. The performance was highest in tests of quantitative and lowest in tasks of spatial skills. Full-blown cognitive skills were already present at the age of four months, and did not significantly change within the investigated time window. The quantitative cross-species comparison showed that, with the exception of spatial skills, the cognitive performance of our birds was on par with those of orang-utans and chimpanzees.

In the following, we will discuss these findings in detail.

### Cognitive performance in physical and social cognitive scales

Overall, we found that our ravens’ physical cognitive performance was very similar to their social cognitive performance, with highest performance scores in quantitative skills and lowest performance scores in spatial skills. These results are not in line with our prediction suggesting that ravens perform differently in the domains of physical and social cognition^48^.

There are several possible explanations. First, differences in physical and social cognitive performance may have simply been obscured by the use of a cognitive test battery designed to tackle potential drivers of human cognitive evolution (see for similar accounts^18,89^). For instance, task design in the PCTB is anchored in the challenges faced by humans and great apes in their daily lives: to find and locate food, use tools and cope with conspecifics. In contrast, although ravens also have to deal with the challenges of discovering and locating food and manoeuvring in a complex social world, they extensively scatter-hoard carcass meat and are non-habitual tool-users^47,90^. The test battery may therefore have not been suitable to pinpoint differences in ravens’ physical and social cognitive skills. However, if this explanation is true, we would have expected to find no differences between scales which does not accord with our observations (but see for a recent study on parrots^56^).

Second, differences in physical and social cognitive performance may only develop later than 16 months of age, and were thus not detected across the four investigated time points. If this explanation were true, we would have expected to find no differences between any tested physical and social cognitive scale across the four different time points, but this was not the case (see Table S4). In addition, recent studies on the development of gaze following skills^77^ and sensorimotor abilities of ravens^72^ showed that the general developmental pace is very fast compared to that of other bird and mammal species.

Third, the assumption that ravens have specialized in the social rather than the physical domain^48^ is simply due to shortage of data. Indeed, due to ravens living in complex societies characterized by fission–fusion dynamics researchers have been fascinated with their social cognitive abilities (see for recent reviews^40,49^). In addition, studies examining single cognitive aspects have provided many crucial aspects to the remarkable tool-kit of ravens’ physical and social cognitive skills (e.g. ^42,46,91,92^). Furthermore, ravens are renowned for caching and hoarding food^40^, combining both sophisticated social (e.g., being highly sensitive to the presence of predators and/or conspecifics that may pilfer caches^40,47^), and physical cognitive skills (such as remembering where and how much food was cached^40, 47^). Hence, our results reveal that ravens are both social and physical intellects, and strengthen recent suggestions that ravens cognitive skills are an expression of general rather than domain specific intelligence^36^.

In addition, a recent reanalysis of the original PCTB dataset of chimpanzees and children^75^ using a confirmatory factor analysis (CFA) did not support the original division of the test battery into a social and a physical cognitive domain. Instead, it identified a spatial cognition factor (see also^93^), suggesting to move beyond the idea that social cognition might be dissociable from physical cognition and evolved separately. The study, thereby, also adds important fuel to the recent debate on cognitive test batteries in animal cognition research (e.g. ^18,56,89^). For instance, some scholars stress to pay more attention to overlooked task demands that may affect performance (e.g., tracking the movement of human experimenters^94^), while others suggest to improve test batteries on multiple fronts such as the design of the tasks, the domains targeted and the species tested^95^. Furthermore, scholars emphasized the importance of addressing the same conceptual question by using tasks that a given species can solve^50^. In addition, Völter and colleagues^96^ proposed a psychometric approach involving a three-step program consisting of (1) tasks that reveal signature limits in performance (i.e. the way individuals make mistakes), (2) assessments of the reliability of individual differences in task performance, and (3) multi-trait multi-method test batteries.

### The development of cognitive skills

The results showed that our ravens’ cognitive performance did not change across the four investigated time points of four, eight, twelve and 16 months respectively. These findings support the prediction that ravens undergo a relatively rapid cognitive development. They further expand recent results on single cognitive skills and sensorimotor development^68,72^ in ravens to the physical cognitive scales of Causalities and Quantities and the manifold domain of social cognition. For instance, Schloegl and colleagues^77^, combining natural observations and behavioural experiments, showed that ravens, shortly after fledging (between 8–15 weeks of age), started to follow the gaze (look where others look) of a conspecific and a human experimenter. This developmental period coincides with ravens still living with their family groups, and the parents still (partially) providing for them. Similarly, studies on two primate species, macaques (*Macaca nemestrina*) and chimpanzees, revealed that individuals of these species started to follow the look-ups of human experimenters at the end of infancy^97,98^. Furthermore, our results are also in line with recent studies on other corvid species linking object permanence abilities to general development. For instance, Pollok and colleagues^67^ showed that magpies master Piagetian Stages 4 and 5 before nutritional independence. Hoffmann and colleagues^99^ investigated whether object permanence abilities are a function of the duration of development across four corvid species. Taking the hatching-to-fledging time as an indicator for development, they showed that Eurasian jays needed by far the shortest time for passing Stage 5 (6 weeks of age) and Stage 6 (7 weeks of age), with carrion crows (Stage 5: 11 weeks of age; Stage 6: 13 weeks of age) and ravens (Stage 5: 11 weeks of age; Stage 6: 14 weeks of age) following several weeks later.

These results are in contrast to findings on individuals of two psittacine species (*Cyanoramphus auriceps, Psittacus erithacus*), which show considerably slower developmental paces and achieve Piagetian Stage 5 only after independence (between 19 weeks of age, respectively 18 weeks of age)^67^. The differences in developmental speed and the linkage to general developmental patterns may reflect a general difference in maturing executive functions and hence cognitive trajectories of corvids and parrots^99^. However, it may also be possible that rapid cognitive development has been selected for in food-storing species, which use memory to retrieve stored food and have a larger hippocampus relative to the rest of the telencephalon than do species that store little or no food^14,59^.

Since ravens’ survival and reproductive output relies heavily on successful cooperation and alliances^40,47^, the rapid pace of ravens’ cognitive toolkit in the physical and social domain may thus also represent a selective response to manoeuvring in a world characterized by the complex challenges of an ever-changing ecological environment and governed by highly cooperative motives^46,47^.

### Comparison of cognitive performance of ravens and great apes

With the exception of spatial skills, the quantitative comparison of performance scores of our ravens and the great ape individuals showed considerable similarities across the two domains of physical and social cognition. These results are also in line with a recent study using the PCTB to test cognitive performance of two Old World monkey species with chimpanzees showing higher performance scores than macaques in tasks of spatial understanding and tool-use only^18^. Since ravens perform impressive flight acrobatics, rely heavily on caching and pilfering of food-stores^40,47^, and have been shown to master stage 6 of object permanence^68^, the relatively low performance scores in the Space scale are surprising. Similarly, a recent study using the PCTB to investigate and compare cognitive skills of four parrot species (*Ara glaucogularis, Ara ambiguus, Primolius couloni, Psittacus erithacus)* showed that the parrots’ performance was also relatively poor in the scale Space (but also across all other scales tested). Individuals were significantly above chance only in the object permanence (*Ara glaucogularis, Primolius couloni, Psittacus erithacus),* and the rotation task (*Ara glaucogularis*^56^. Hence, our findings may echo Köhler who noted that “the success of the intelligence tests in general will be more likely endangered by the person making the experiment than by the animal” (p 265^100^). Since, ravens’ and other corvids’ social life is highly competitive^101^, all aspects of their cognitive abilities have likely been shaped by the need to out-compete conspecifics in general. It thus may be possible that our ravens’ performance in the scale Space—but also all other physical cognitive scales—was overshadowed by a social component with the ravens perceiving the experimenter as a competitor for the food reward. These findings may add a new aspect to proposals suggesting to integrate a competitive component into experimental designs^71,102^.

In contrast to our ravens’ performance, however, the parrots tested by Krasheninnikova and colleagues^56^ performed at chance level across all three physical and all three social cognitive scales. These results are in stark contrast to previous findings on parrots’ remarkable cognitive capacities (see for reviews^49,103^). They also  emphasize Tinbergen’s notion that the same test for a different species may therefore not be the same test^104^. Furthermore, differences in test performance between individuals of the parrot and our study may also be due to differences in socialization such as hand-raising, habituation and training procedures, and social bond strength between the birds and the experimenters (see also^77,105^). For instance, the birds in the present study were tested by two highly familiar people who had also hand-raised them. In contrast, tests in the study of Krasheninnikova and colleagues^56^ had been conducted by ten familiar experimenters, which had not hand-raised them, and four unfamiliar assistants. Hence, future studies should investigate the impact of these factors on cognitive performance in more detail to minimize possible counterproductive effects. In addition, analyses of why species fail in certain tests in combination with informed accounts of their ecological and social validity will aid in getting a better understanding of whether distinct tasks are too easy or too difficult for a given species to be solved^18,89,102^.

Furthermore, it is certainly an issue that the test battery was constructed and administered by humans^10^, influencing cognitive performance of our ravens overall. For instance, Schloegl and colleagues^77^ investigated the ontogeny of gaze following in ravens by using observations of spontaneously occurring gaze following behaviour between conspecifics and controlled experiments involving human experimenters. They found that visual co-orientation with conspecifics emerged around eight weeks of age, while gaze following behaviour to human-given cues could only be observed seven weeks later. Schloegl and colleagues^82^ suggested that human models may not be capable of providing the same stimulus quality as a conspecific due to emphasizing different aspects for eliciting gaze following behaviour. In contrast, Heinrich^47^ suggested that there is something unique about ravens that permits an uncanny closeness to develop with humans, thereby allowing insights in skills that could otherwise never be discovered.

Taken together, the present experiments provide evidence that our ravens’ experimental performance was on par with those of adult great apes in the similar tasks. They thus strengthen the idea that ravens evolved a general and flexible neural system for higher cognition^36,106^ rather than being highly specialized in a few domains only^107^. Yet, we do not claim that the cognitive abilities of ravens and great apes are generally similar since similarity at the behavioural level does not need to reflect the same underlying cognitive mechanisms^50^. This may be particular true for complex cognitive abilities such as tool use, cooperation, or referential signalling that involve different cognitive building blocks^36^. For example, referential signalling may involve aspects of learning, memory, empathy, and theory of mind, but the degree to which each of the abilities are involved and has advanced may differ between species and taxonomic groups^46,108,109^. In addition, it may also be the case that the cognitive competencies in the items tested in the PCTB simply did not differ substantially^18^. Furthermore, proponents of situated cognition argue that cognition reaches beyond the brain and tackle the relation between cognitive processes, on the one hand, and their neuronal, bodily, and worldly basis, on the other (for a review see^110^). This means that choices made via non-homologous body parts—beaks (ravens), hands (great apes), and eyes (ravens) combining panoramic sight with excellent stereoscopic vision^111^—not only involve different effectors but also different processors possibly influencing cognitive processing and output.

In addition, we do not claim that the cognitive performance of our eight ravens can be generalized to the species as a whole and corvids in general. For instance, some random effects seem to have influenced task performance suggesting to pay special attention in future studies to personality, task-performance across age and thus ontogeny of test-subjects (see e.g.^112^). Hence, the present study may pave the way to future collaborative studies and data sharing across research labs encouraging a *ManyBirds* project (see for related efforts^113,114^). It may thus aid in 1) tackling one of the biggest obstacles in Animal Cognition research, to obtain sufficient sample sizes, and 2) improving and adapting distinct tasks of test-batteries to better implement and mimic the ecology of the respective model species (see also^115,116^). Therefore, future studies should expand the range of investigated skills in a given test-battery beyond social interactions with humans and foraging contexts, and situate the findings within a comparative evolutionary framework (see also^95,96,116^). Furthermore, we hope to inspire more research into the impact of ontogeny on cognitive performance, which, although constituting one of Tinbergen’s four why’s, is especially lagging behind in studies of Animal Cognition^117,118^.

## Conclusion

Here, we systematically tested the physical and social cognitive skills of eight hand-raised ravens, members of the corvid family, with a special focus on development. The results enabled the first direct, quantitative comparison with the cognitive performance of individuals of two great ape species, chimpanzee and orangutans, tested across the same domains and tasks. Our results suggest that ravens are not only social intellects but have also developed sophisticated cognitive skills for dealing with the physical world. Furthermore, their cognitive development was very rapid and their cognitive performance was on par with adult great apes’ cognitive performance across the same cognitive scales. Our findings thus put recent assessments of ravens’ and great apes’ conspicuous similarities in single cognitive paradigms on solid footing. In addition, they show that the impact of ecological challenges of species’ cognitive development has, at least in the field of cognition, been severely underestimated and that socialization may influence test performance. Hence, studying cognition requires also an understanding of the dynamic of the different influences that, during ontogeny, contributes to adult cognition^118^.

## Supplementary information

Supplementary Video 1.

Supplementary Video 2.

Supplementary Video 3.

Supplementary Video 4.

Supplementary Video 5.

Supplementary Video 6.

Supplementary Video 7.

Supplementary Tables.

Supplementary Video 8.

Supplementary Video 9.

Supplementary Video 10.

Supplementary Video 11.

Supplementary Video 12.

Supplementary Video 13.

Supplementary Video 14.

Supplementary Video 15.

## References

[1] Shettleworth SJ (2009). Cognition, Evolution, and Behavior.

[2] McMillan N, Hahn AH, Spetch ML, Sturdy CB (2015). Avian cognition: examples of sophisticated capabilities in space and song. Wiley Interdiscip. Rev. Cogn. Sci..

[3] Bshary R, Wickler W, Fricke H (2002). Fish cognition: a primate's eye view. Anim. Cogn..

[4] Olkowicz S (2016). Birds have primate-like numbers of neurons in the forebrain. Proc. Natl. Acad. Sci..

[5] Jarvis ED (2005). Avian brains and a new understanding of vertebrate evolution. Nat. Rev. Neurosci..

[6] van Schaik CP (2003). Orangutan cultures and the evolution of material culture. Science.

[7] Krupenye C, Kano F, Hirata S, Call J, Tomasello M (2016). Great apes anticipate that other individuals will act according to false beliefs. Science.

[8] Fröhlich M (2016). Unpeeling the layers of language: Bonobos and chimpanzees engage in cooperative turn-taking sequences. Sci. Rep..

[9] Amici F, Aureli F, Call J (2010). Monkeys and apes: are their cognitive skills really so different?. Am. J. Phys. Anthropol..

[10] Herrmann E, Call J, Hernandez-Lloreda MV, Hare B, Tomasello M (2007). Humans have evolved specialized skills of social cognition: the cultural intelligence hypothesis. Science.

[11] Wobber V, Herrmann E, Hare B, Wrangham R, Tomasello M (2014). Differences in the early cognitive development of children and great apes. Dev. Psychobiol..

[12] Spearman C (1904). 'General Intelligence', objectively determined and measured. Am. J. Psychol..

[13] Tomasello M, Carpenter M, Call J, Behne T, Moll H (2005). Understanding and sharing intentions: the origins of cultural cognition. Behav. Brain Sci..

[14] Jolly A (1966). Lemur social behaviour and primate intelligence. Science.

[15] R. W. Byrne, A. Whiten, Eds., *Machiavellian Intelligence: Social Expertise and the Evolution of Intellect in Monkeys, Apes and Humans* 413 (Clarendon Press, Oxford, 1988).

[16] Seyfarth RM, Cheney DL (2017). Precursors to language: Social cognition and pragmatic inference in primates. Psychon. Bull. Rev..

[17] Dunbar RIM, Shultz S (2007). Evolution in the social brain. Science.

[18] Schmitt V, Pankau B, Fischer J (2012). Old World monkeys compare to apes in the primate cognition test battery. PLoS ONE.

[19] Milton, K. In *Machiavellian Intelligence: Social Expertise and the Evolution of Intellect in Monkeys, Apes and Humans,* (eds Byrne, R. W., & Whiten, A.) 285–305 (Clarendon Press, Oxford, 1988)

[20] DeCasien AR, Williams SA, Higham JP (2017). Primate brain size is predicted by diet but not sociality. Nat. Ecol. Evol..

[21] González-Forero M, Gardner A (2018). Inference of ecological and social drivers of human brain-size evolution. Nature.

[22] Humphrey, N. K. In *Growing**Points**in Ethology *(eds Bateson, P. P. G. & Hinde, R. A.) 303–317 (Cambridge University Press, Cambridge, 1976).

[23] Kudo H, Dunbar R (2001). Neocortex size and social network size in primates. Anim. Behav..

[24] Street SE, Navarrete AF, Reader SM, Laland KN (2017). Coevolution of cultural intelligence, extended life history, sociality, and brain size in primates. Proc. Natl. Acad. Sci..

[25] Lodato S, Arlotta P (2015). Generating neuronal diversity in the mammalian cerebral cortex. Annu. Rev. Cell Dev. Biol..

[26] Chittka L, Niven J (2009). Are bigger brains better?. Curr. Biol..

[27] Herculano-Houzel S (2017). Numbers of neurons as biological correlates of cognitive capability. Curr. Opin. Behav. Sci..

[28] Healy SD, Rowe C (2007). A critique of comparative studies of brain size. Proc. R. Soc. B Biol. Sci..

[29] Scheiber IBR (2008). Does ‘relationship intelligence’make big brains in birds?. Open Biol. J..

[30] Emery NJ, Seed AM, von Bayern AMP, Clayton NS (2007). Cognitive adaptations of social bonding in birds. Philos. Trans. R. Soc. B Biol. Sci..

[31] McComb K, Moss C, Durant SM, Baker L, Sayialel S (2001). Matriarchs as repositories of social knowledge in African elephants. Science.

[32] Holekamp KE (2007). Questioning the social intelligence hypothesis. Trends Cogn. Sci..

[33] Clayton NS, Emery NJ (2015). Avian models for human cognitive neuroscience: a proposal. Neuron.

[34] Emery NJ, Clayton NS (2004). The mentality of crows: Convergent evolution of intelligence in corvids and apes. Science.

[35] Jarvis ED (2014). Whole-genome analyses resolve early branches in the tree of life of modern birds. Science.

[36] Güntürkün O, Bugnyar T (2016). Cognition without cortex. Trends Cogn. Sci..

[37] Kuenzel WJ, Medina L, Csillag A, Perkel DJ, Reiner A (2011). The avian subpallium: new insights into structural and functional subdivisions occupying the lateral subpallial wall and their embryological origins. Brain Res..

[38] Güntürkün, O., Stacho, O. & Ströckens, F. In *Evolution of Nervous Systems 2e,* Vol. 1 (ed. Kaas, J.) 171–221 (Elsevier, Oxford, 2017).

[39] Stacho, M. *et al.* A cortex-like canonical circuit in the avian forebrain. *Science* **369**, eabc5534 (2020). 10.1126/science.abc5534 32973004

[40] Bugnyar T (2013). Social cognition in ravens. Comp. Cogn. Behav. Rev..

[41] Clayton NS, Dickinson A (1998). Episodic-like memory during cache recovery by scrub jays. Nature.

[42] Kabadayi C, Osvath M (2017). Ravens parallel great apes in flexible planning for tool-use and bartering. Science.

[43] Hunt GR, Gray RD (2004). The crafting of hook tools by wild New Caledonian crows. Proc. R. Soc. Lond. B.

[44] Bird CD, Emery NJ (2009). Insightful problem solving and creative tool modification by captive nontool-using rooks. Proc. Natl. Acad. Sci. U. S. A. (PNAS).

[45] Taylor AH, Hunt GR, Medina FS, Gray RD (2009). Do New Caledonian crows solve physical problems through causal reasoning?. Proc. R. Soc. B Biol. Sci..

[46] Pika S, Bugnyar T (2011). The use of referential gestures in ravens (*Corvus corax*) in the wild. Nat. Commun..

[47] Heinrich B (1991). The Mind of the Raven: Investigations and Adventures with Wolf-Birds.

[48] Massen JJM, Pašukonis A, Schmidt J, Bugnyar T (2014). Ravens notice dominance reversals among conspecifics within and outside their social group. Nat. Commun..

[49] Lambert ML, Jacobs I, Osvath M, von Bayern AMP (2019). Birds of a feather? Parrot and corvid cognition compared. Behaviour.

[50] Seed AM, Emery NJ, Clayton NS (2009). Intelligence in corvids and apes: a case of convergent evolution?. Ethology.

[51] Schloegl C, Kotrschal K, Bugnyar T (2008). Do common ravens (*Corvus corax*) rely on human or conspecific gaze cues to detect hidden food?. Anim. Cogn..

[52] Seed AM, Emery NJ, Clayton NS (2006). Investigating physical cognition in rooks, *Corvus frugilegus*. Curr. Biol..

[53] Bugnyar T, Reber SA, Buckner C (2016). Ravens attribute visual access to unseen competitors. Nat. Commun..

[54] Jacobs IF, Osvath M (2015). The string-pulling paradigm in comparative psychology. J. Comp. Psychol..

[55] Shettleworth SJ (2010). Clever animals and killjoy explanations in comparative psychology. Trends Cogn. Sci..

[56] Krasheninnikova A, Berardi R, Lind M-A, O’Neill L, von Bayern AMP (2019). Primate cognition test battery in parrots. Behaviour.

[57] MacLean EL, Herrmann E, Suchindran S, Hare B (2017). Individual differences in cooperative communicative skills are more similar between dogs and humans than chimpanzees. Anim. Behav..

[58] F. Antinucci, In *'Language' and Intelligence in Monkeys and Apes: Comparative Developmental Perspectives* (eds Parker, S. T. & Gibson, K. R.) 157–171 (Cambridge University Press, Cambridge, UK, 1994).

[59] Clayton NS (1992). The ontogeny of food-storing and retrieval in marsh tits. Behaviour.

[60] Gómez J-C (2005). Species comparative studies and cognitive development. Trends Cogn. Sci..

[61] Mills W (1898). The Nature and Development of Animal Intelligence.

[62] Morgan CL (1896). Habit and Instinct.

[63] Tinbergen N (1963). On aims and methods in ethology. Zeitschrift für Tierpsychologie.

[64] Piaget J (1954). The Construction of Reality in the Child.

[65] Davidson G, Miller R, Loissel E, Cheke LG, Clayton NS (2017). The development of support intuitions and object causality in juvenile Eurasian jays (Garrulus glandarius). Sci. Rep..

[66] Zucca P, Milos N, Vallortigara G (2007). Piagetian object permanence and its development in Eurasian jays (*Garrulus glandarius*). Anim. Cogn..

[67] Pollok B, Prior H, Güntürkün O (2000). Development of object permanence in food-storing magpies (*Pica pica*). J. Comp. Psychol..

[68] Bugnyar T, Stöwe M, Heinrich B (2007). The ontogeny of caching in ravens, *Corvus corax*. Anim. Behav..

[69] Pepperberg IM, Funk MS (1990). Object permanence in four species of psittacine birds: an African Grey parrot (*Psittacus erithacus*), an Illiger mini macaw (Ara maracana), a parakeet (*Melopsittacus undulatus*), and a cockatiel (*Nymphicus hollandicus*). Anim. Learn. Behav..

[70] Funk MS (1996). Development of object permanence in the New Zealand parakeet (*Cyanoramphus auriceps*). Anim. Learn. Behav..

[71] Pepperberg IM (1999). The Alex Studies, Cognitive and Communicative Abilities of Grey Parrots.

[72] Jacobs I, Kabadayi C, Osvath M (2019). The development of sensorimotor cognition in common ravens (*Corvus corax*) and its comparative evolution. Anim. Behav. Cogn..

[73] Warneken F, Tomasello M (2006). Altruistic helping in human infants and young chimpanzees. Science.

[74] Ladygina-Kohts, N. N. *Infant Chimpanzee and Human Child. A Classic 1935 Comparative Study of Ape Emotions and Intelligence*. 592 (Oxford University Press, New York, 1935).

[75] Herrmann E, Hernández-Lloreda MV, Call J, Hare B, Tomasello M (2010). The structure of individual differences in the cognitive abilities of children and chimpanzees. Psychol. Sci..

[76] Sima MJ (2018). Doctoral Thesis.

[77] Schloegl C, Kotrschal K, Bugnyar T (2007). Gaze following in common ravens, *Corvus corax*: Ontogeny and habituation. Anim. Behav..

[78] Baayen RH, Davidson DJ, Bates DM (2008). Mixed-effects modeling with crossed random effects for subjects and items. J. Mem. Lang..

[79] McCullagh, P. & Nelder, J. A. *Generalized Linear Models*. (CRC Press, Boca Raton, 1989), vol. 37.

[80] Baayen RH (2008). Analyzing Linguistic Data. A Practical Introduction to Statistics Using R.

[81] Barr DJ, Levy R, Scheepers C, Tily HJ (2013). Random effects structure for confirmatory hypothesis testing: keep it maximal. J. Mem. Lang..

[82] Schielzeth H, Forstmeier W (2008). Conclusions beyond support: overconfident estimates in mixed models. Behav. Ecol..

[83] Matuschek H, Kliegl R, Vasishth S, Baayen H, Bates D (2017). Balancing Type I error and power in linear mixed models. J. Mem. Lang..

[84] Dobson AJ, Barnett A (2011). An Introduction to Generalized Linear Models.

[85] Field, A. *Discovering Statistics Using SPSS*. (Sage Publications, 2005).

[86] Fox J, Monette G (1992). Generalized collinearity diagnostics. J. Am. Stat. Assoc..

[87] R Development Core Team, A language and environment for statistical computing. R Foundation for Statistical Computing, Vienna, Austria. Retrieved from https://www.R-project.org. (2011).

[88] D. Bates *et al.*, Package ‘lme4’. *convergence* **12**, 1 (2015).

[89] MacLean EL (2012). How does cognition evolve? Phylogenetic comparative psychology. Anim. Cogn..

[90] Heinrich B (1988). Raven tool use?. The Condor.

[91] Heinrich B, Bugnyar T (2005). Testing problem solving in ravens: string-pulling to reach food. Ethology.

[92] Boeckle M, Bugnyar T (2012). Long-term memory for affiliates in ravens. Curr. Biol..

[93] Hopkins WD, Russell JL, Schaeffer J (2014). Chimpanzee intelligence is heritable. Curr. Biol..

[94] Jelbert SA, Taylor AH, Gray RD (2016). Does absolute brain size really predict self-control? Hand-tracking training improves performance on the A-not-B task. Biol. Let..

[95] Shaw RC, Schmelz M (2017). Cognitive test batteries in animal cognition research: evaluating the past, present and future of comparative psychometrics. Anim. Cogn..

[96] Völter CJ, Tinklenberg B, Call J, Seed AM (2018). Comparative psychometrics: establishing what differs is central to understanding what evolves. Philos. Trans. R. Soc. B Biol. Sci..

[97] Ferrari PF, Kohler E, Fogassi L, Gallese V (2000). The ability to follow eye gaze and its emergence during development in macaque monkeys. Proc. Natl. Acad. Sci..

[98] Tomasello M, Hare B, Fogleman T (2001). The ontogeny of gaze following in chimpanzees, Pan troglodytes, and rhesus macaques, Macaca mulatta. Anim. Behav..

[99] Hoffmann A, Rüttler V, Nieder A (2011). Ontogeny of object permanence and object tracking in the carrion crow, Corvus corone. Anim. Behav..

[100] Koehler W (1925). The Mentality of Apes.

[101] Marzluff JM, Angell T (2005). In the Company of Crows and Ravens.

[102] Hare B (2001). Can competitive paradigms increase the validity of experiments on primate social cognition?. Anim. Cogn..

[103] van Horik J, Emery NJ (2011). Evolution of cognition. WIREs Cogn. Sci..

[104] Tinbergen N (1951). The Study of Instinct.

[105] Péron F, Rat-Fischer L, Nagle L, Bovet D (2010). ‘Unwilling’versus ‘unable’: do grey parrots understand human intentional actions?. Interact. Stud..

[106] Balakhonov D, Rose J (2017). Crows rival monkeys in cognitive capacity. Sci. Rep..

[107] Mendes N, Hanus D, Call J (2007). Raising the level: Orangutans use water as a tool. Biol. Let..

[108] Watson SK (2015). Vocal learning in the functionally referential food grunts of chimpanzees. Curr. Biol..

[109] Vail AL, Manica A, Bshary R (2013). Referential gestures in fish collaborative hunting. Nat. Commun..

[110] Cheng K (2018). Cognition beyond representation: Varieties of situated cognition in animals. Comp. Cogn. Behav. Rev..

[111] Güntürkün, O. Sensory physiology: Vision. *Sturkies avian physiology*, 1–19 (2000).

[112] Miller R, Laskowski KL, Schiestl M, Bugnyar T, Schwab C (2016). Socially driven consistent behavioural differences during development in common ravens and carrion crows. PLoS ONE.

[113] Bohn,M. *et al.* ManyPrimates. Open Sci. *Framework*, (2018).

[114] Frank, M. C. The manybabies project. See https://manybabies.github.io/(Accessed 12 July *2018*), (2015).

[115] Kamil AC (1987). A synthetic approach to the study of animal intelligence. Nebr. Symp. Motiv..

[116] Bräuer J, Hanus D, Pika S, Grey R, Uomini N (2020). Old and new approaches to animal cognition: there is not “one cognition”. J. Intell..

[117] Bateson P, Laland KN (2013). Tinbergen's four questions: an appreciation and an update. Trends Ecol. Evol..

[118] Boesch, C. Mothers, environment, and ontogeny affect cognition. *Anim. Behav. Cogn.* **7**(3), 474–489 (2020).

