# Supplementary material for: Ravens parallel great apes in physical and social cognitive skills
Source: Sci Rep. 2020 Dec 10;10:20617. doi: 10.1038/s41598-020-77060-8 (PMC7728792; doi:10.1038/s41598-020-77060-8)
Supplement: Supplementary file 8 — Supplementary Tables. [file 41598_2020_77060_MOESM8_ESM.pdf]

Supplementary Materials for

Ravens parallel great apes in physical and social cognitive skills

Simone Pika*, Miriam Jennifer Sima, Christian Blum, Esther Herrmann,

and Roger Mundry

*Correspondence should be addressed to
Simone Pika: spika@uos.de

This PDF file includes:

Apparatuses
Tables S1 to S8

Apparatuses
First round (4 months of age)

Open Bottle. In the open bottle task, food pieces ("Frolic") were inserted in an open plastic bottle without a lid and the bottle was laid down horizontally. The bird had to lift the bottle and tilt it to access the reward.

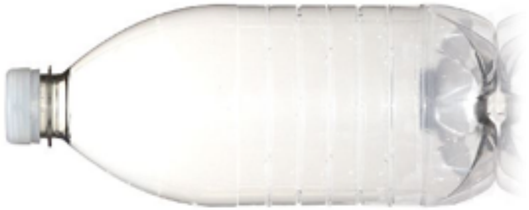

String pulling. The apparatus consisted of four different wooden boxes, which all had a hemp string attached that could be pulled. A reward was inserted in the wooden box at the far-right end and the bird had the possibility to retrieve the food by pulling the connected string. If the bird succeeded in this baseline condition without any demonstration of the experimenter, a second reward was inserted in the inner right box to raise the level of difficulty (Here, the bird had to first pull at the first string and then the second string additionally to retrieve the reward.).

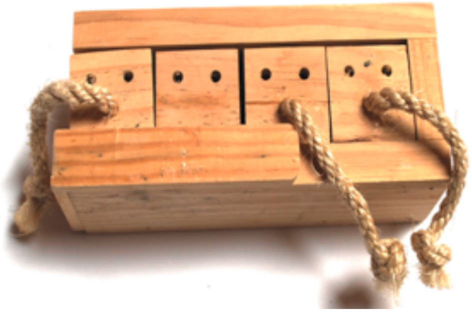

Seesaw. The apparatus consisted of a transparent mobile tube fixed to a wooden seesaw. The tube had a lid on one side. A reward could only be obtained by tilting the seesaw in one specific direction (the one without the lid). The reward was placed in the middle of the tube.

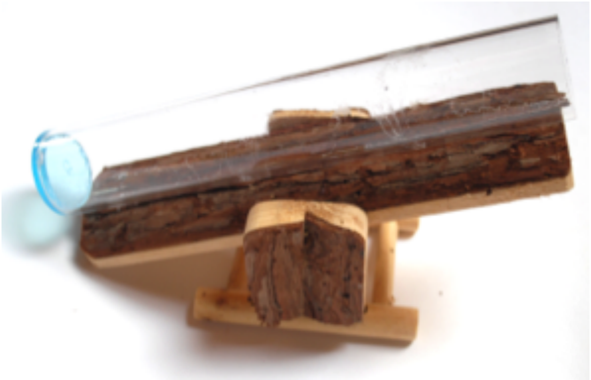

Second round (8 months of age)

Board game. This apparatus consisted of a tube being loosely attached to a wooden board with two cable ties. The reward was placed in the tube. The bird had to peck against the tube to detach it from the cable ties and to retrieve the reward.

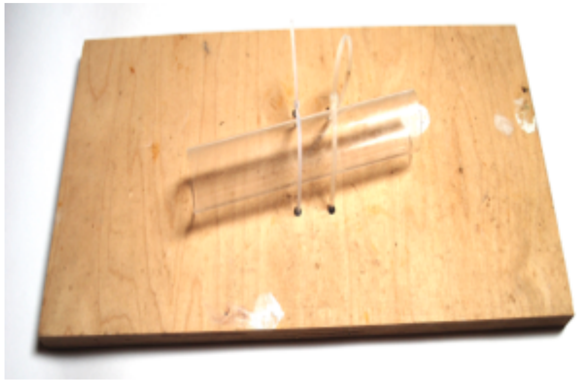

Turntable. The apparatus consisted of two connected, planar rotatable press boards with a hole per press board. The experimenter placed a reward in the hole and rotated the top plate. To retrieve the reward, the bird had to peck against the hole of the top plate.

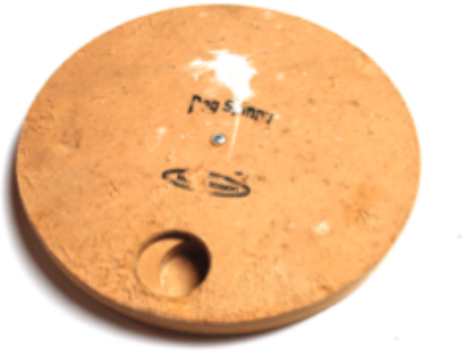

Activity Board. The apparatus consisted of several plastic tokens including different possibilities to hide a reward and different covering and closing mechanisms (e.g., pulling, pushing).

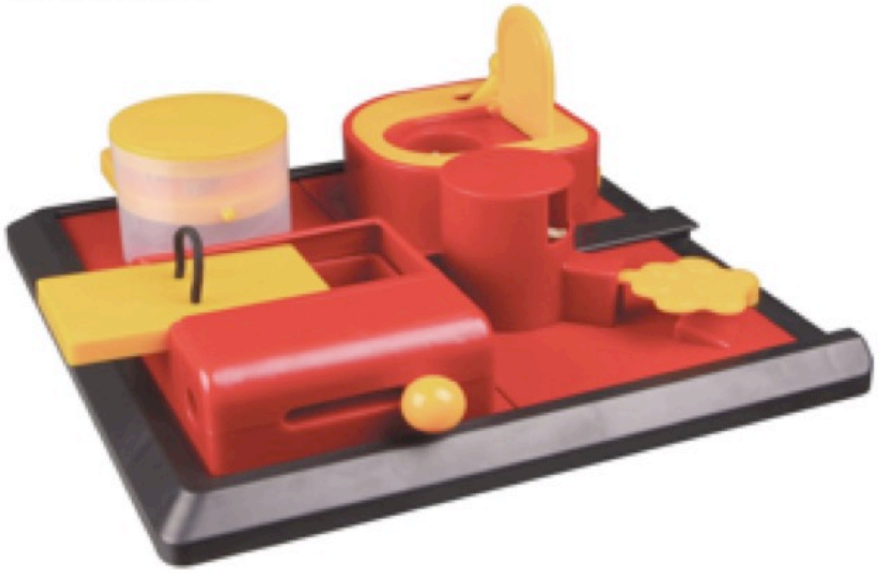

Activity Board - Sliding. The experimenter placed a reward in the red box with the yellow slider and closed the slider. To retrieve the reward, the bird had to pull the slider. All other possible tasks on the activity board were not baited and were also not touched by the experimenter.

Third round (12 months of age)

Activity Board – Bone lever. In this task, the experimenter placed the reward in the red cylinder with the yellow lid and closed the lid. To retrieve the food, the bird had to peck/push the lever.

Activity Board – Paw. In this task the experimenter placed the reward in the opening of the red tower. The bird had to step/push the yellow paw-like shape to lift and retrieve the food.

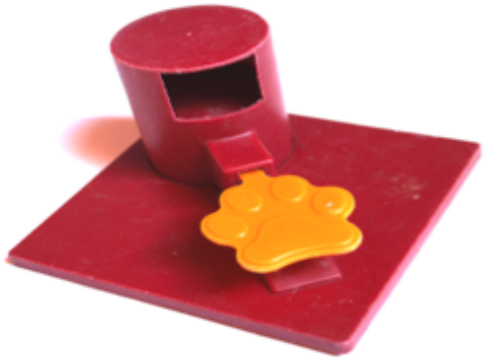

Activity Board – Folding door. In this task, the experimenter opened the yellow lid to place the food on the yellow plate inside. To drop and retrieve the reward, the bird had to pull the yellow plate out of the transparent cylinder.

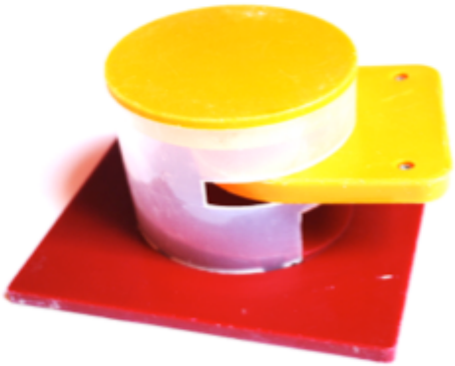

Fourth round (16 months of age)

Pull and find. This apparatus consisted of a wooden block with a hole to the right and a hole to the right side to hide a reward. Both holes could be covered by pegs (a red and a blue one), which were connected to a yellow pole in the middle of the wooden block. The experimenter placed a

reward in one of the two holes and placed then the respective peg on the hole with the reward. To retrieve the reward, the bird had to pull the string on the respective side.

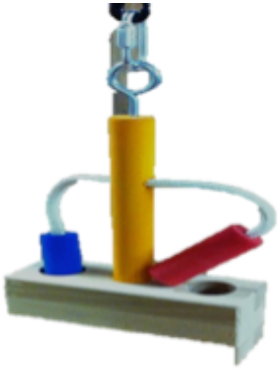

Turn and find. This apparatus consisted of a wooden block with a hole, a hole to hide a reward, a blue plug and a red lid. The experimenter baited the hole and moved the red lid to the side to cover the reward. Then the experimenter placed the plug to fix the lid. To retrieve the reward, the bird had to remove the plug and turn the lid to access the hole with the reward.

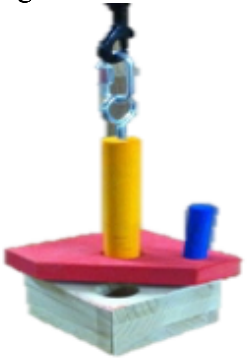

Open the lid. This apparatus consisted of four opaque lids which all could be opened in different ways. For this task, we only used the box which is marked with an x. The experimenter baited the box (marked with an x) and closed the lid. To retrieve the reward, the bird had to lift the lid by pulling at the lid.

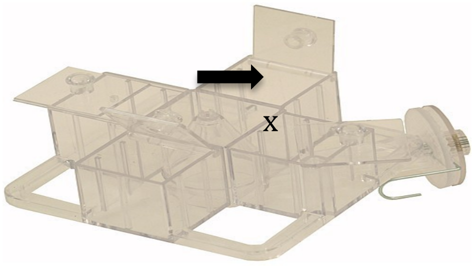

Table S1. Results of the full model addressing the interaction between cognitive performance across different scales and age (estimates, together with standard errors, confidence limits, significance tests as well as minimum and maximum of model estimates obtained after excluding levels of random effects one at a time).

Term	Estimate	SE	lower Cl	upper Cl	Chisq	df	p	min	max
Intercept	0.995	0.131	0.748	1.265				0.881	1.080
Age[(1)]	0.003	0.110	-0.193	0.198				-0.151	0.050
Scale Communication	0.028	0.097	-0.160	0.214				-0.026	0.127
Scale Quantity	0.247	0.102	0.048	0.462				0.133	0.310
Scale Space	-0.349	0.083	-0.509	-0.187				-0.426	-0.255
Scale ToM	-0.038	0.125	-0.278	0.232				-0.170	0.069
Sex[(2)]	0.011	0.079	-0.141	0.165	0.020	1	0.889	-0.127	0.081
Experimenter[(3)]	-0.257	0.204	-0.686	0.117	1.570	1	0.210	-0.369	-0.033
Age:Scale Communication[(4)]	0.034	0.157	-0.291	0.338	2.417	4	0.660	-0.034	0.163
Age:Scale Quantities[(4)]	-0.164	0.178	-0.527	0.191				-0.269	0.028
Age:Scale Space[(4)]	-0.095	0.161	-0.385	0.176				-0.161	0.147
Age:Scale ToM[(4)]	-0.256	0.212	-0.652	0.123				-0.370	0.125

(1) z-transformed to a mean of zero and a standard deviation of one; mean and sd of the original age were 42.877 and 21.940 weeks, respectively

(2) dummy coded with female being the reference category

(3) dummy coded factor with two levels

(4) the reference category was 'Causality'; the indicated test refers to the overall effect of the interaction

Table S2. Results of the full model with regard to the random effects (estimated standard deviation).

Grouping variable	Effect[1]	Sd
Olre	Intercept	<0.001
Item	Experimenter.M	0.156
item	Sex.male	<0.001
item	z.Age_weeks	<0.001
item	Intercept	0.004
Task	Experimenter.M	0.482
task	Sex.male	<0.001
task	z.Age_weeks	<0.001
task	Intercept	<0.001
Individual	z.Age_weeks:scale.Theory.of.Mind	<0.001
Individual	z.Age_weeks:scale.Space	0.160
Individual.2	z.Age_weeks:scale.Quantity	<0.001
Individual.3	z.Age_weeks:scale.Communication	<0.001
Individual.4	scale.Theory.of.Mind	<0.001
Individual.5	scale.Space	<0.001
Individual.6	scale.Quantity	<0.001
Individual.7	scale.Communication	0.073
Individual.8	Experimenter.M	0.128
Individual.9	z.Age_weeks	0.046
Individual.10	Intercept	<0.001
Sibling_Group	z.Age_weeks:scale.Theory.of.Mind	<0.001
Sibling_Group.1	z.Age_weeks:scale.Space	<0.001
Sibling_Group.2	z.Age_weeks:scale.Quantity	<0.001
Sibling_Group.3	z.Age_weeks:scale.Communication	0.076
Sibling_Group.4	scale.Theory.of.Mind	<0.001
Sibling_Group.5	scale.Space	0.069
Sibling_Group.6	scale.Quantity	<0.001
Sibling_Group.7	scale.Communication	<0.001
Sibling_Group.8	Experimenter.M	0.116
Sibling_Group.9	Sex.male	<0.001

Sibling_Group.10	z.Age_weeks	<0.001
Sibling_Group.11	Intercept	0.090

(1) 'intercept' depicts to a random intercept, all others to a random slope

Table S3. Results of the reduced model (lacking the interaction between age and scale) addressing differences in physical and social cognitive performance and ontogenetic trajectories in ravens.

Term	Estimate	SE	Chisq	Df	p
Intercept	0.982	0.121			
Age(1)	-0.063	0.062	1.005	1	0.316
Scale Communication(2)	0.035	0.095	13.836	4	0.008
Scale Quantity(2)	0.247	0.101			
Scale Space(2)	-0.347	0.082			
Scale ToM(2)	-0.073	0.121			
Sex(3)	0.011	0.079	0.019	1	0.891
Experimenter(4)	-0.228	0.181	1.591	1	0.207

(1) z-transformed to a mean of zero and a standard deviation of one; mean and sd of the original age were 42.877 and 21.940 weeks, respectively

(2) the reference category was 'Causality'; the indicated test refers to the overall effect of the interaction

(3) dummy coded with female being the reference category

(4) dummy coded factor with two levels

Table S4. Results of the pairwise comparison of scales addressing ontogenetic trajectories in ravens. The numbers depicted in **bold** show significant results, the numbers depicted in *italics* show trends.

Reference level	Comparison with	Estimate	SE	z	p
Causality	Communication	0.035	0.095	0.367	0.714
Causality	Quantity	**0.247**	**0.101**	**2.439**	**0.015**
Causality	Space	**-0.347**	**0.082**	**-4.245**	**<0.001**
Causality	Theory of Mind	-0.073	0.121	-0.603	0.547
Communication	Causality	-0.040	0.092	-0.430	0.668
Communication	Quantity	*0.213*	*0.113*	*1.883*	*0.060*
Communication	Space	**-0.383**	**0.096**	**-3.984**	**<0.001**
Communication	Theory of Mind	-0.106	0.131	-0.810	0.418
Quantity	Causality	**-0.252**	**0.102**	**-2.482**	**0.013**
Quantity	Communication	*-0.212*	*0.116*	*-1.830*	*0.067*
Quantity	Space	**-0.595**	**0.105**	**-5.690**	**<0.001**
Quantity	Theory of Mind	**-0.319**	**0.139**	**-2.300**	**0.021**
Space	Causality	**0.349**	**0.075**	**4.680**	**<0.001**
Space	Communication	**0.390**	**0.094**	**4.146**	**<0.001**
Space	Quantity	**0.600**	**0.099**	**6.053**	**<0.001**
Space	Theory of Mind	**0.284**	**0.121**	**2.349**	**0.019**

Table S5. Results of the random effects for the full model addressing species difference in performance as a function of scale (estimated standard deviation).

Grouping variable	Effect[(1)]	Sd
Olre	Intercept	<0.001
task.in.ind	Intercept	<0.001
Individual	scale.Theory.of.Mind[(2)]	<0.001
Individual	scale.Space[(2)]	0.382
Individual	scale.Quantities[(2)]	<0.001
Individual	scale.Communication[(2)]	0.091
Individual	Intercept	0.150
Item	Sex.male[(3)]	<0.001
Item	Species.Chimp[(4)]	0.301
Item	Species.Orang[(4)]	.069
Item	Intercept	0.380
Task	Sex.male[(3)]	<0.001
Task	Species.Chimp[(4)]	0.354
Task	Species.Orang[(4)]	0.300
Task	Intercept	<0.001

(

[1)] 'intercept' depicts to a random intercept, all others to a random slope
[(2)] dummy coded; the reference category was Causality
[(3)] dummy coded with female being the reference category
[(4)] dummy coded, the reference category was Raven

Table S6. Results of the random effects for the full model addressing species difference in performance as a function of scale for those tasks for which chance probability was unknown (estimated standard deviation).

Grouping variable	Effect[(1)]	Sd
Olre	Intercept	0.707
Individual	scale.Theory.of.Mind[(2)]	1.378
Individual	scale.Social.Learning[(2)]	0.939
Individual	Intercept	0.748
Item	Sex.male[(3)]	<0.001
Item	Species.Orang[(4)]	<0.001
Item	Species.Chimp[(4)]	1.013
Item	Intercept	0.666
Grp	Effect	Sdcor
Olre	Intercept	0.707

[(1)] 'intercept' depicts to a random intercept, all others to a random slope
[(2)] dummy coded; the reference category was Causality
[(3)] dummy coded with female being the reference category
[(4)] dummy coded, the reference category was Raven

Table S7. Results of the full model addressing species differences in the performance as a function of scale.

ref.scale	Term	Estimate	Std. Error
Causality	Intercept[(1)]	0.875	0.224
Causality	spec.relChimp[(2)]	0.374	0.255
Causality	spec.relOrang[(2)]	0.259	0.217
Causality	scaleCommunication[(3)]	-0.025	0.355
Causality	scaleQuantities[(3)]	0.227	0.411
Causality	scaleSpace[(3)]	-0.533	0.321
Causality	scaleTheory of Mind[(3)]	-0.080	0.447
Causality	Sex.male[(4)]	0.049	0.043
Causality	spec.relChimp:scaleCommunication	0.133	0.401
Causality	spec.relOrang:scaleCommunication	0.253	0.327
Causality	spec.relChimp:scaleQuantities	-0.065	0.421
Causality	spec.relOrang:scaleQuantities	-0.170	0.320
Causality	spec.relChimp:scaleSpace	1.139	0.369
Causality	spec.relOrang:scaleSpace	0.666	0.317
Causality	spec.relChimp:scaleTheory of Mind	-0.142	0.497
Causality	spec.relOrang:scaleTheory of Mind	-0.149	0.406
Communication	Intercept[(1)]	0.875	0.224
Communication	spec.relChimp[(2)]	0.373	0.255
Communication	spec.relOrang[(2)]	0.258	0.217
Communication	scaleCommunication[(3)]	-0.027	0.353
Communication	scaleQuantities[(3)]	0.227	0.411
Communication	scaleSpace[(3)]	-0.533	0.321
Communication	scaleTheory of Mind[(3)]	-0.080	0.447
Communication	Sex.male[(4)]	0.049	0.043
Communication	spec.relChimp:scaleCommunication	0.134	0.399
Communication	spec.relOrang:scaleCommunication	0.254	0.325
Communication	spec.relChimp:scaleQuantities	-0.064	0.420
Communication	spec.relOrang:scaleQuantities	-0.170	0.319
Communication	spec.relChimp:scaleSpace	1.139	0.369

Communication	spec.relOrang:scaleSpace	0.666	0.317
Communication	spec.relChimp:scaleTheory of Mind	-0.142	0.497
Communication	spec.relOrang:scaleTheory of Mind	-0.149	0.406
Quantities	Intercept(1)	0.875	0.223
Quantities	spec.relChimp(2)	0.374	0.255
Quantities	spec.relOrang(2)	0.259	0.217
Quantities	scaleCommunication(3)	-0.025	0.354
Quantities	scaleQuantities(3)	0.227	0.411
Quantities	scaleSpace(3)	-0.533	0.320
Quantities	scaleTheory of Mind(3)	-0.080	0.447
Quantities	Sex.male(4)	0.049	0.043
Quantities	spec.relChimp:scaleCommunication	0.133	0.401
Quantities	spec.relOrang:scaleCommunication	0.253	0.327
Quantities	spec.relChimp:scaleQuantities	-0.064	0.420
Quantities	spec.relOrang:scaleQuantities	-0.170	0.319
Quantities	spec.relChimp:scaleSpace	1.139	0.369
Quantities	spec.relOrang:scaleSpace	0.666	0.317
Quantities	spec.relChimp:scaleTheory of Mind	-0.142	0.497
Quantities	spec.relOrang:scaleTheory of Mind	-0.149	0.406
Space	Intercept(1)	0.854	0.210
Space	spec.relChimp(2)	0.394	0.239
Space	spec.relOrang(2)	0.281	0.198
Space	scaleCommunication(3)	-0.021	0.346
Space	scaleQuantities(3)	0.226	0.401
Space	scaleSpace(3)	-0.463	0.283
Space	scaleTheory of Mind(3)	-0.079	0.435
Space	Sex.male(4)	0.044	0.043
Space	spec.relChimp:scaleCommunication	0.129	0.387
Space	spec.relOrang:scaleCommunication	0.247	0.314
Space	spec.relChimp:scaleQuantities	-0.063	0.406
Space	spec.relOrang:scaleQuantities	-0.169	0.305
Space	spec.relChimp:scaleSpace	1.057	0.330

Space	spec.relOrang:scaleSpace	0.591	0.267
Space	spec.relChimp:scaleTheory of Mind	-0.142	0.478
Space	spec.relOrang:scaleTheory of Mind	-0.149	0.388
Theory of Mind	Intercept(1)	0.875	0.224
Theory of Mind	spec.relChimp(2)	0.374	0.255
Theory of Mind	spec.relOrang(2)	0.259	0.217
Theory of Mind	scaleCommunication(3)	-0.025	0.355
Theory of Mind	scaleQuantities(3)	0.227	0.411
Theory of Mind	scaleSpace(3)	-0.533	0.321
Theory of Mind	scaleTheory of Mind(3)	-0.080	0.447
Theory of Mind	Sex.male(4)	0.049	0.043
Theory of Mind	spec.relChimp:scaleCommunication	0.133	0.401
Theory of Mind	spec.relOrang:scaleCommunication	0.253	0.327
Theory of Mind	spec.relChimp:scaleQuantities	-0.064	0.421
Theory of Mind	spec.relOrang:scaleQuantities	-0.170	0.320
Theory of Mind	spec.relChimp:scaleSpace	1.139	0.369
Theory of Mind	spec.relOrang:scaleSpace	0.666	0.317
Theory of Mind	spec.relChimp:scaleTheory of Mind	-0.142	0.497
Theory of Mind	spec.relOrang:scaleTheory of Mind	-0.149	0.407

(1) 'intercept' depicts to a random intercept, all others to a random slope
(2) dummy coded, the reference category was Raven
(3) dummy coded; the reference category was Causality
(4) dummy coded with female being the reference category

Table S8. Proportion of the correct performance of the three species across the different cognitive scales.

	**Chimpanzee**	**Orang-utan**	**Raven**	**Chance probability[1]**
Causality	0.636	0.617	0.543	0.5
Quantity	0.676	0.625	0.597	0.5
Space	0.664	0.504	0.369	0.35
Communication	0.496	0.459	0.512	0.5
Social Learning	0.097	0.073	0.046	-
Theory of Mind	0.365	0.323	0.343	0.5

[1] Chance probability considers only those tasks for which it could be determined but proportion correct is based on all tasks.
